# Selector function of MHC I molecules is determined by protein plasticity

**DOI:** 10.1038/srep14928

**Published:** 2015-10-20

**Authors:** Alistair Bailey, Neil Dalchau, Rachel Carter, Stephen Emmott, Andrew Phillips, Jörn M. Werner, Tim Elliott

**Affiliations:** 1 Institute for Life Sciences, Building 85, University of Southampton, SO17 1BJ, UK; 2 Computational Science Laboratory, Microsoft Research, 21 Station Road, Cambridge, CB1 2FB, UK; 3 Cancer Sciences Unit, Faculty of Medicine, University of Southampton, Southampton, SO16 6YD, UK; 4 Centre for Biological Sciences, Faculty of Natural & Environmental Sciences, Building 85, University of Southampton, SO17 1BJ, UK

## Abstract

The selection of peptides for presentation at the surface of most nucleated cells by major histocompatibility complex class I molecules (MHC I) is crucial to the immune response in vertebrates. However, the mechanisms of the rapid selection of high affinity peptides by MHC I from amongst thousands of mostly low affinity peptides are not well understood. We developed computational systems models encoding distinct mechanistic hypotheses for two molecules, HLA-B*44:02 (B*4402) and HLA-B*44:05 (B*4405), which differ by a single residue yet lie at opposite ends of the spectrum in their intrinsic ability to select high affinity peptides. We used *in vivo* biochemical data to infer that a conformational intermediate of MHC I is significant for peptide selection. We used molecular dynamics simulations to show that peptide selector function correlates with protein plasticity, and confirmed this experimentally by altering the plasticity of MHC I with a single point mutation, which altered *in vivo* selector function in a predictable way. Finally, we investigated the mechanisms by which the co-factor tapasin influences MHC I plasticity. We propose that tapasin modulates MHC I plasticity by dynamically coupling the peptide binding region and α_3_ domain of MHC I allosterically, resulting in enhanced peptide selector function.

Peptides bound to *Major histocompatibility complex class I* (MHC I) molecules are displayed at the surface of most nucleated cells in jawed vertebrates for surveillance by *cytotoxic T-lymphocytes* (CTL)^1^,^2^,^3^. In transformed or diseased cells, peptides derived from viral or aberrantly expressed proteins are presented alongside peptides derived from native proteins. This is possible because MHC I has degenerate specificity: each molecule can bind a range of peptides of different lengths and sequences. In selecting peptides and presenting them at the cell surface, MHC I provides CTLs with a sample of the internal cell proteome. This property makes MHC I an attractive target for the development of immunotherapies that exploit the CTL response. Examples include therapies that modulate the overall cell surface presentation of peptides by MHC I, or that target MHC I at the cell surface with specific peptide vaccines to make cells more, or less, visible to CTLs.

The selection of peptides by MHC I occurs in the *endoplasmic reticulum* (ER) and is modulated by a multi-protein complex into which the MHC I:β_2_m heterodimer is recruited. This *peptide loading complex* comprises of the peptide *Transporter associated with Antigen Presentation* (TAP), which transports peptides into the ER, chaperone proteins calreticulin and ERp57, and an MHC I-specific cofactor *tapasin* (reviewed in^4^). Incorporation of MHC I molecules into the peptide loading complex locates them in close proximity to the peptide supply. Here, MHC I interacts with cofactor molecules to preferentially select peptides of high affinity from the large intracellular pool of many potential peptides of largely lower affinity^5^,^6^. Selection of high affinity peptides confers stability and immunogenicity to MHC I^7^,^8^ and is one of the most important factors in establishing the specificity and intensity of a CTL response^9^. We refer to the *peptide selector function* of MHC I as its ability to preferentially select higher affinity peptides over lower affinity ones. This in turn profoundly influences the downstream MHC I function of presenting peptides to CTLs.

MHC I molecules are highly polymorphic, but have a common tertiary structure (Fig. 1) consisting of: the polymorphic heavy chain, monomorphic β_2_-microglobulin (β_2_m), and a peptide of generally 8–10 amino acids in length, non-covalently bound into a peptide binding groove. Intrinsic differences in the peptide selector function of different allelic variants of MHC I molecules become most apparent when the co-factor molecule tapasin is non-functional or absent. This is because tapasin masks these intrinsic differences by preferentially enhancing the selector function of MHC I molecules that are inefficient at selecting high affinity peptides^10^,^11^. When tapasin is absent, differences in the primary sequence of MHC I are sufficient to alter peptide selector function^12^. For example, two human alleles HLA-B*35:01 and HLA-B*52:01 differ by 12 residues in their primary sequence. In the absence of tapasin, HLA-B*35:01 molecules are expressed at a high level at the cell surface, whilst HLA-B*52:01 molecules are only observed at low levels^13^. Remarkably, even a single amino acid difference can alter the intrinsic peptide selector function of MHC I: HLA-B*44:02 (B*4402) and HLA-B*44:05 (B*4405) differ by a single residue at position 116 (Fig. 1A), yet they lie at opposite ends of the spectrum in their intrinsic ability to select high affinity peptides in the absence tapasin^10^. In tapasin-deficient cells, B*4402 is poor at sampling the peptidome and as a consequence is degraded in the endoplasmic reticulum (ER), while B*4405 is able to effectively select high affinity peptides and present them at the cell surface.

These observations of allelic differences in intrinsic MHC I peptide selector function imply that tapasin normalizes the peptide selector function of MHC I alleles. However, a mechanistic explanation for how tapasin achieves this is lacking. Furthermore, despite there being many crystal structures of MHC I, the structural basis for intrinsic differences in selector function between MHC I alleles remains unknown. Importantly, what is not revealed by X-ray crystallography is the formation of a MHC I peptide complex and the significance or otherwise of the processes involved. For example, the structures of B*4402 and B*4405 reveal highly similar peptide bound conformations with a RMSD of 0.3 Å between their secondary structures^15^,^16^ (Fig. 1B), and are therefore unable to provide insight into the known functional differences in peptide selection. However, the simple observation that the peptide is buried deep in the peptide binding groove of MHC I (Fig. 1C) suggests that MHC I is likely to explore intermediate conformational states during the formation of a stable complex, simply to allow peptides to enter and leave the groove. In other words, this observation alone suggests that MHC I molecules must be plastic in order to sample and bind a range of peptides. Different methods have provided indirect evidence that peptide binding to MHC I is associated with a conformational intermediate^17^,^18^,^19^,^20^,^21^, including molecular dynamics simulations^22^,^23^,^24^,^25^. Although these studies have helped to resolve the apparent paradox of how MHC I molecules of such degenerate specificity can bind peptides with such high affinity, they do not provide a framework for understanding the mechanism of peptide selection inside cells.

Our aim therefore was to further elucidate the relationship between MHC I structure and MHC I function in the context of the complexity of the cellular environment. We sought to address this challenge for the MHC I presentation system, by adopting an interdisciplinary approach to infer mechanism from *in vivo* biochemical experiments. We use computational systems models that test several mechanistic hypotheses of MHC I peptide selection, and quantify the uncertainty in each model. This provides us with a rigorous mathematical basis for comparing competing mechanistic hypotheses, and for identifying which hypothesis best explains the experimental data. As becomes apparent below, in the case of antigen processing this centers on the plasticity of MHC I. We then relate observations at the cellular level to protein structure and function using molecular dynamics simulations of MHC I. As has been noted, combining three disciplines presents challenges to the reader in interpreting the results and assessing their validity^26^. However, this approach yields information about mechanism that only becomes apparent when considering all three disciplines in combination. Specifically, we test the hypothesis that conformational intermediates of MHC I molecules are directly relevant for the peptide selector function of MHC I. This work represent a first step towards establishing a general framework for inferring molecular mechanism from complex biological data in the context of MHC I peptide selection, with its subsequent importance for modulating the immune response.

## Results

### An enhanced intrinsic selector function for B*4405 over B*4402

To investigate the mechanisms of peptide selection by MHC I, we first quantified peptide selector function *in vivo* for two functionally distinct MHC I molecules, B*4402 and B*4405. We then developed computational systems models of MHC I peptide selection as sets of biochemical reactions to encode distinct mechanistic hypotheses, and used Bayesian model selection to determine which hypotheses were most likely, given the experimental data.

**Figure 2:**
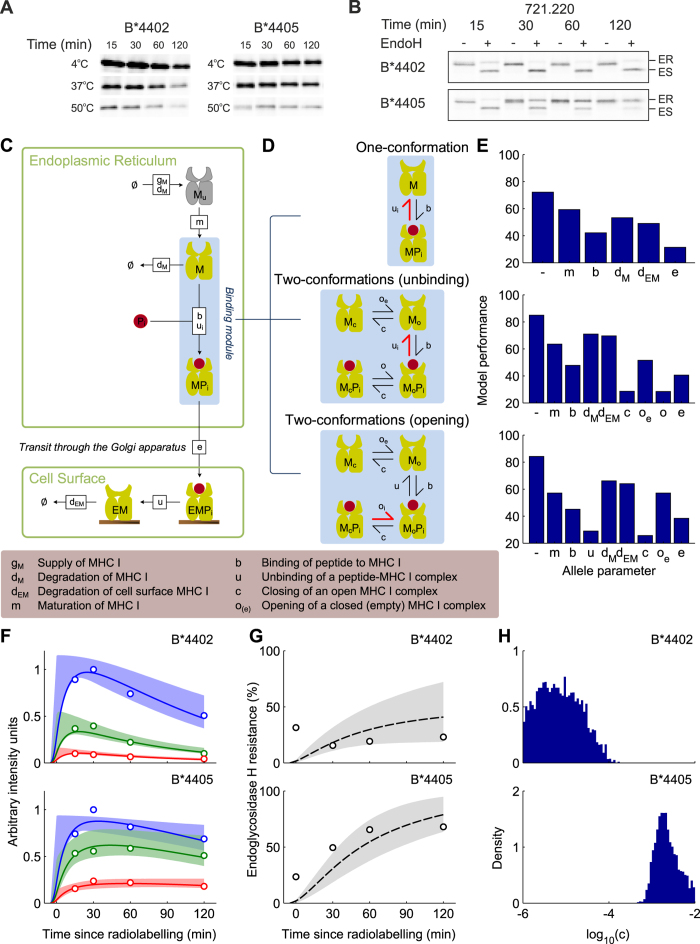
Computational systems models of the mechanisms of MHC I peptide selection, fit to *in vivo* peptide selection data for B*4402 and B*4405 in the absence of tapasin. (**A**) Time-dependent peptide selection measured with pulse-chase and thermostability in tapasin-deficient cells, as described in materials and methods. Tapasin deficient cultured 220 cells (721.220) were metabolically radiolabelled with ^35^S-met for 5 min, then chased for the indicated times before being lysed and heated to the temperatures shown (data quantified in F). (**B**) Cell surface transit measured as percentage endoglycosidase-H (EndoH) resistance. MHC I was immunoprecipitated with W6/32 and treated at the different time points with (+) or without (−) EndoH, which distinguishes between sensitive, pre-cis-Golgi (ES) and resistant (ER) post-cis-Golgi MHC I (data quantified in G). Immunoprecipitations were performed in such a way as to record only MHC I bound to high affinity peptides (*10*). (**C**) A *general* computational systems model of the mechanisms of peptide selection in the absence of tapasin. Shapes represent molecular species and labelled boxes represent reactions and their rate parameters. (**D**) Different binding mechanisms are illustrated for o*ne-conformation* and *two-conformation* models, where peptide-dependent reactions are indicated by thick red symbols. (**E**) Comparison of model performance for different parameters taking allele-specific values (*allele parameters*). The most likely model (lowest BIC score) was the two-conformation model with MHC I opening as the peptide-dependent step and MHC I closing rate *c* as the allele-dependent parameter. (**F**,**G**) Comparison of the most likely model (solid lines) against experimental measurements (circles), with 95% confidence intervals (shaded regions). (**F**) Quantification of pulse chase experiments shown in panel A (circles), together with model simulations (lines). Red indicates the proportion of MHC I molecules that remained after heating to 50 °C (high affinity peptide-MHC complexes), green indicates heating to 37 °C (medium and high affinity complexes) and blue indicates heating to 4 °C (all complexes). (**G**) Quantification of cell surface transit experiments shown in panel B (circles), together with model simulations (lines). (**H**) Marginal posterior density of the allele-specific closing rate *c*, reflecting the probability of the parameter values, conditional on the measured data and underlying model.

To quantify peptide selector function *in vivo*, we measured the fraction of a pulse-labelled cohort of MHC I molecules that were stable at 50 °C, 37 °C and 4 °C as they progressed through the secretory pathway over 120 minutes (Fig. 2A,B, quantified in F,G). Since the thermal stability of MHC I has been shown to act as a surrogate for the affinity of peptide bound to MHC I^10^, this allows the quantification of the selection of high affinity peptides by MHC I. Figure 2A shows that, as demonstrated previously^10^, in tapasin-deficient cells B*4405 is able to select high affinity peptides. This is indicated by the relative proportions of thermostable MHC I complexes harvested from the cells at each time point.

Over time, B*4405 (Fig. 2A, quantified in F) has a larger proportion of complexes stable at higher temperatures. However, B*4402, with a lower proportion of stable complexes was therefore less able to select high affinity peptides. Furthermore, in the absence of tapasin, B*4402 is mostly sensitive to the enzyme Endoglycosidase H which indicates that B*4402 was less able to acquire high affinity peptides and progress to the cell surface (Fig. 2B, quantified in G).

### A conformational intermediate of MHC I is significant for peptide selector function

To investigate the mechanisms of MHC I peptide selection, we developed computational systems models of the antigen processing pathway as sets of biochemical reactions that encoded distinct mechanistic hypotheses. We defined our models as extensions to a previous computational systems model of MHC I assembly, peptide selection and cell surface presentation^27^. This modelling approach enabled us to observe the effects of individually or simultaneously varying all parameters over physiologically relevant ranges. It also allowed testing of different mechanistic hypotheses against biological data, thereby providing a springboard for further experimental investigation. Our previous analysis identified the rate of peptide binding to MHC I as the parameter differentiating HLA-B alleles B*4402, B*4405, and B*2705^27^. To determine how the rate of peptide binding might be controlled by MHC I itself, in addition to factors such as the concentration of peptide in the ER or the volume of the ER, we incorporated inherent plasticity of MHC I as a minimal extension to the original model^27^, by explicitly describing two conformational states of MHC I: a peptide-receptive *open* conformation to which peptides bind at rate *b*, and a non-receptive *closed* conformation (Fig. 2C,D). We represented MHC I and peptide as a system of molecular species that interact with defined reaction rates, including rates of MHC I and peptide generation and degradation, and transport from the ER to the cell surface. The reaction rates were considered as unknown parameters, and were fitted to the experimental data (as described in the Supplementary Information). In these extended models, the release of peptides from MHC I was described in two steps: MHC I opening, followed by peptide-unbinding. This gave rise to two variant models in which the interactions determined by the biochemical affinity of peptide for MHC I, the *peptide-dependent step*, could arise from one of two processes. One being that the rate of MHC I opening (*o*_*i*_) is peptide-dependent, the other being that the rate of peptide-unbinding from the open MHC I conformation (*u*_*i*_) is peptide-dependent. The subscript *i* indicates the opening or unbinding rate for a given peptide *i*. The descriptor “open” does not have a measured structural correlate, but we assumed that the structural correlate of “closed” was the common crystallographic structure of peptide bound MHC I complexes. This assumption includes MHC I containing very weakly bound peptides such as those that occupy only the F-pocket^28^.

To determine which mechanistic hypotheses were most likely, we compared model simulations with experimental data using Bayesian parameter inference and model selection. To simulate the range of thermostability that was measured, representative peptides with high, medium or low affinities for MHC I were used in the model. In Fig. 2F,G, the experimental data from Fig. 2A,B is indicated by circles, and the model simulations reproducing the data are indicated by lines. To assess the plausibility of each model, we determined the optimal parameter values using probabilistic inference techniques^27^. We examined a variety of hypotheses for allele-specific mechanisms, by enabling specific parameters (such as the rate of closing) to take on different values for each allele, while keeping the remaining parameters the same for all alleles^27^. For each hypothesis, we refer to the variable parameter as the *allele parameter*. Conclusions were based on the Bayesian Information Criterion (BIC)^29^, which minimizes the deviation between model simulation and experimental observation, while accounting for the effects of having different numbers of free parameters in the different models that were tested. Figure 2E shows the BIC statistic (shorter bars indicate statistical improvement) for a range of allele parameters for each of the three models, including no allele parameter. Comparing BIC statistics for the one conformational model (Fig. 2E, top) indicated that an allele-specific rate of peptide binding to MHC I (*b*) explains the differences in peptide selection between the three alleles better than an allele-specific rate of peptide free MHC I degradation (*d*_*M*_). Importantly, we found that both of the two-conformation models outperformed the original one-conformation model in their ability to reproduce the experimental data, with improved performance sufficient for their additional parameters to be considered justifiable by the BIC statistic (Fig. 2E). In other words, this analysis clearly indicates that when testing model hypotheses of peptide selection, a two-conformation MHC I model provides a better explanation of the experimental observations than a one-conformation model. In particular, the one-conformation model was unable to reproduce a key experimental observation of slow conversion of MHC complexes to a mature measurable state, which had a maximal signal at 30 minutes post radiolabelling (see Figure S7). Moreover, the two-conformation model where the opening rate (*o*_*i*_) of the closed MHC I peptide complex (M_c_P_i_) was peptide-dependent, and the closing rate (*c*) of open peptide bound MHC I (M_o_P_i_) was allele-dependent, provided the best description of the data (Fig. 2E, bottom).

### Faster closing of MHC I corresponds with enhanced intrinsic selector function

Interestingly, for the most likely model determined in Fig. 2E, B*4405 showed a relatively fast closing rate (Fig. 2H, bottom) corresponding with faster progression to a stable conformation, consistent with its enhanced intrinsic selector function observed experimentally (Fig. 2F, bottom). B*4402 showed a slower closing rate (Fig. 2H, top), corresponding with a slower progression to a stable conformation, consistent with a diminished intrinsic selector function (Fig. 2F,G, top). Our results therefore suggest that the intrinsic differences in selector function between these MHC I alleles arise from intrinsic differences in their ability to move from open to closed conformations. The closing rate *c* corresponds to the rate at which MHC I progresses to a stable conformation, from which it is able to exploit the peptide-dependent opening step. Specifically, frequent progression to a stable conformation (i.e. fast closing) enables the preferential exchange of low affinity peptides (characterized by fast *o*_*i*_) for high affinity peptides (characterized by slow *o*_*i*_), thus enhancing selector function. Put simply, once an MHC I molecule binds a peptide and closes, it is the peptide-dependent opening rate of the MHC I molecule that defines the affinity of the peptide-MHC interaction. Therefore, if one MHC I molecule closes faster than another, the faster closing MHC I molecule reaches the peptide-dependent part of the selection process more quickly, resulting in enhanced peptide selector function due to more rapid sampling of peptides.

### Peptide selector function of MHC I correlates with protein plasticity

Following the identification of functionally relevant conformations of MHC I, we investigated the extent to which these conformations could be linked to protein plasticity. Specifically, we performed molecular dynamics simulations of MHC I alleles B*4402^16^ and B*4405^15^, and quantified their plasticity by computing the range and frequency of molecular conformations, as described by the relative motions of their backbone atoms, under conditions of thermodynamic equilibrium. We then compared the plasticity of these alleles to their corresponding peptide selector function.

We performed molecular dynamics simulations using the GROMACS package^30^ in the peptide bound and peptide free states (see Supplementary Information Table S1 for a summary of the simulations). The simulations were designed to capture the dynamic behavior of each MHC I molecule in a manner independent of timescale. Specifically, we used 10 ns block averaged root mean squared fluctuations to assess that there was adequate configurational sampling during the 420 ns simulations, to be consistent with equilibrium behavior (Figure S1).

**Figure 3:**
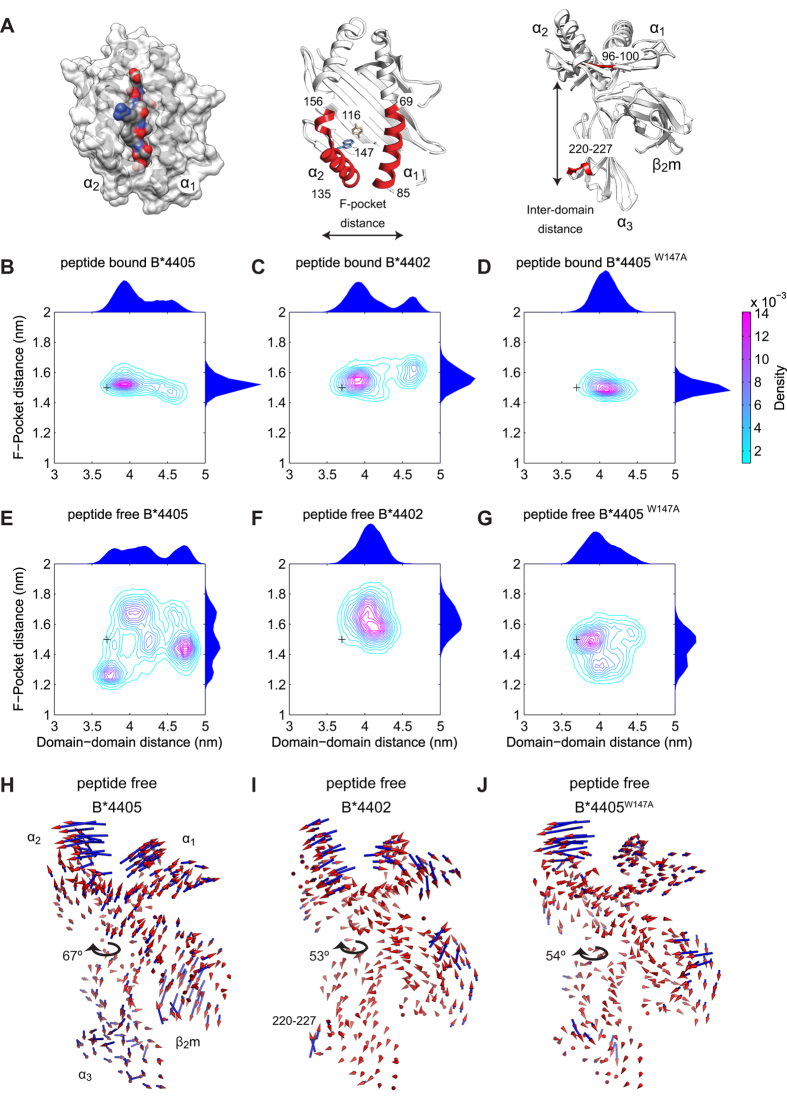
Quantification of protein plasticity for MHC I alleles B*4402, B*4405 and B*4405^W147A^ from molecular dynamics simulations. (**A**) Left: Surface representation of peptide bound MHC I. Middle: Ribbon representations of peptide free MHC I. The polymorphism between B*4402 and B*4405 at position 116 in the peptide binding groove (brown) and mutation B*4405^W147A^ (blue). F-pocket distances were measured between the center of mass of helix residues 135–156 and 69–85 (red). Right: Inter-domain distances were measured between peptide binding groove residues 96–100 (red) and α_3_ residues 220–227 (red). (**B–G**) Contour plots of the joint probability densities for the conformations of MHC I populated in each simulated condition, as defined by distances in (**A**). Black crosses indicate the initial structure conformation. Distributions for each individual distance are plotted on the outside of the adjacent axis. (**H–J**) The motion most correlated with the distance fluctuations across the F-pocket as defined in (**A**). Cones indicate the direction and amplitude of motion. The range of inter-domain twisting for each molecule is indicated by arrows (as depicted in Figure S5). See also Figures S1–S5.

To quantify the range and frequency of conformations adopted by MHC I molecules, we chose to focus on two representative distance measures, the *F-pocket distance* and the *inter-domain distance*. The F-pocket distance corresponds to the distance between the center of mass of helix residues 135–156 and helix residues 69–85 (Fig. 3A, middle). This was chosen due to the hinge points created between the break in the α_2_ helix near residue 156 and the end of the α_2_ helix near residue 135, about which conformational change may occur^23^. Thus, the F-pocket distance was indicative of the range of conformations that could be adopted by the peptide binding groove. The inter-domain distance corresponds to the distance between peptide binding groove residues 96–100 and α_3_ domain residues 220–227 (Fig. 3A, right). This was chosen to represent the range of motion that could be exhibited between the heavy chain domains. From the molecular dynamics simulations, we quantified the frequency with which the MHC I molecules adopted conformations with a given F-pocket and inter-domain distance, represented as joint probability distributions (Fig. 3B,C,E,F). These distributions indicate the conformations that MHC I could adopt, together with the correlations between peptide binding groove and heavy chain conformations.

To characterize how motion of the peptide binding groove relates to overall motion of MHC I, we performed Functional Mode Analysis (FMA) (See Supplementary Information and Figure S3 for further details)^31^. In correlating the F-pocket distance to the collective motions of the backbone atoms of the molecule, FMA determined a single collective motion most correlated with the distance fluctuations across the F-pocket, represented as porcupine plots (Fig. 3H,I). The direction of movement in the plots is represented by the orientation of the cones, while the amplitude of movement is represented by both the size and color of the cones. In this way, FMA provides a description of the conformational change undergone by the whole MHC I molecule corresponding to a conformational change of the peptide binding groove.

We then compared the plasticity of MHC I alleles B*4402 and B*4405 (Fig. 3) with their corresponding peptide selector function (Fig. 2). In the peptide bound state (Fig. 3B,C), both molecules displayed similar plasticity and populated a single dominant conformation, with a sub-population of conformations arising from inter-domain motion for both alleles. Since these two alleles differ by only a single amino acid and exhibit almost identical crystal structures in their peptide bound state, we sought to investigate whether the observed differences in peptide selector function were due to differences in protein plasticity in the peptide free state. When we repeated the simulations following removal of the peptide, this revealed significant differences in plasticity (Fig. 3E,F). In the absence of peptide, B*4402 populated a single F-pocket conformation (Fig. 3F). In contrast, B*4405 populated several F-pocket conformations (Fig. 3E) and explored greater conformational space overall. In Fig. 3H,I, the direction and amplitude of the backbone atoms indicated a twisting motion that correlated with F-pocket opening, articulated around the domain linker region. This twisting motion was significantly larger for B*4405 than for B*4402 (see Figure S5 for more details of the twisting analysis).

Taken together, the molecular conformations and movements of atoms indicated that, in the absence of peptide, the F-pocket of B*4402 was less plastic than that of B*4405, exploring a more limited conformational space and exhibiting a reduced twisting motion (Fig. 3I; Figure S5). Furthermore, these differences in plasticity in the absence of peptide correlated with differences in peptide selector function, with the more plastic B*4405 exhibiting significantly greater selector function than the less plastic B*4402 (Fig. 2F,G). Interestingly, this plasticity also correlated with the rate of conformational change from an open to a closed conformation in the computational systems model, given by the closing rate *c*, with B*4405 exhibiting a significantly greater closing rate than B*4402. Therefore, by comparing quantifications of B*4405 and B*4402 plasticity (Fig. 3) with *in vivo* measurements of peptide selection (Fig. 2F,G), we identified mechanistic hypotheses linking protein plasticity to peptide selector function, as targets for further investigation.

### Plasticity of MHC I can predict peptide selector function *in vivo*

To test our hypotheses experimentally, we performed molecular dynamics simulations of a site-directed mutant of B*4405 in which the highly conserved Tryptophan 147 was changed to Alanine (B*4405^W147A^).The identification of this novel mutant was somewhat fortuitous and stemmed from a previous study^32^ describing the effect of 33 random single site mutations on the recognition of HLA-A*02:01 by alloreactive T cells. During this study, a significant effect of a W147 mutation on MHC I intracellular trafficking was observed, suggesting this site as a potential target for investigation. The B*4405^W147A^ novel mutant revealed an interesting phenotype that enabled an investigation into the link between sequence, plasticity and selector function. Significantly, in peptide free molecular dynamics simulations, B*4405^W147A^ explored fewer peptide binding groove conformations than wild-type B*4405 (Fig. 3G), and also showed a corresponding reduction in the range of the inter-domain twisting, similar to peptide-free B*4402 dynamics (Fig. 3J; Figure S5). Furthermore, B*4405^W147A^ showed mobility of the α_3_ domain more like B*4402 than B*4405 (Fig. 3J), including a highly dynamic 220–227 α_3_ loop. Together, these simulations predict that the intrinsic peptide selector function of B*4405^W147A^ should be qualitatively similar to that of B*4402.

**Figure 4:**
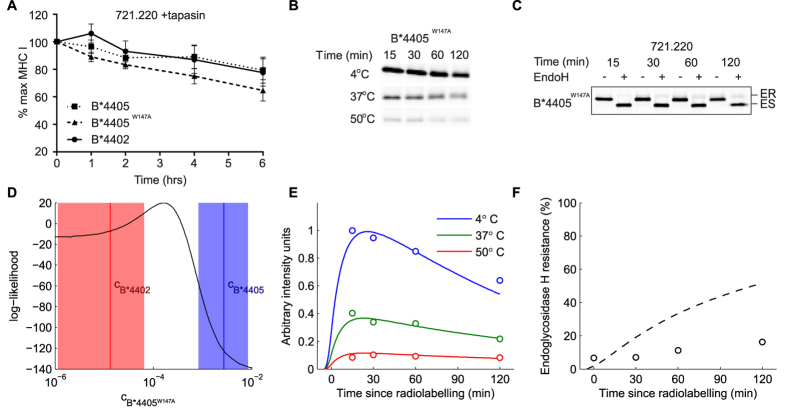
Peptide selection of B*4405^W147A^ measured *in vivo* and compared with simulations of computational systems model. (**A**) Mutant B*4405^W147A^ has similar peptide binding ability to that of B*4402 and B*4405. This is demonstrated by performing a BFA decay assay with 220. tapasin cell lines expressing each allele. Stability of peptide loaded MHC I over time is measured with the conformation specific antibody W6/32. (**B,C**) Pulse-chase thermostability and EndoH assays in the absence of tapasin were carried out for B*4405^W147A^ as in Fig. 2A,B, and as described in materials and methods. (**D**) Combined likelihood score against data for all three alleles in absence of tapasin, for different values of the allele parameter c. An optimum for B*4405^W147A^ is present between the mean posterior values for the other two alleles, as labelled. The areas indicate the 95% confidence intervals for those two parameters. (Middle, Right) simulation values for the maximum likelihood value of c. (**E**,**F**) Simulation (lines) of the two-conformation (opening) model with allele parameter *c* set to the value in panel (**D**) that best fits the experimental data (i.e. that optimizes the likelihood function). Plotted together with experimental measurements of B*4405^W147A^ (circles). (**E**) Experiments relate time-dependent peptide selection measured with pulse-chase and thermostability in tapasin-deficient cells (quantification of panel B). Red symbols/lines indicate heating to 50 °C (corresponding to high affinity peptide-MHC complexes), green indicates 37 °C (medium and high affinity complexes) and blue indicates 4 °C (all complexes). (**F**) Cell surface transit measured as percentage EndoH resistance (quantification of panel (**C**)).

Having tested that B*4405^W147A^ molecules were able to select and bind to peptides normally (Fig. 4A), we tested our prediction by performing pulse chase analysis as in Fig. 2 for the B*4405^W147A^ mutant (Fig. 4B,C). We found that the W147A mutation of B*4405 profoundly affected the ability of MHC I to select stable peptides and present them at the cell surface in the absence of tapasin, as predicted. To mechanistically model the selector function of this mutant, we fit the allele parameter c (closing rate), using the optimal two-conformation model of Fig. 2. The allele parameter for B*4405^W147A^ fell between the parameter values for B*4405 and B*4402 (Fig. 4D), resulting in a peptide selector function close to that of B*4402 (compare Fig. 4E,F with 2F,G), as predicted.

### Restraint of the tapasin binding site can modulate MHC I protein plasticity

To further investigate our hypothesis that protein plasticity determines selector function, we sought to identify potential mechanisms that could be used by chaperones to regulate protein plasticity. Specifically, we focused on the MHC I allele B*4402 and the chaperone molecule tapasin, which has been shown to significantly alter peptide selector function. Previous work identified a C-terminus domain residue of tapasin, R333, which interacts with a residue of the 220–227 α_3_ loop, E222, in docking simulations between HLA-B*08:01 and tapasin^4^. Within this region, mutation of position 222 has been shown to abrogate the interaction between MHC I and tapasin, leading to loss of function^33^. Furthermore, polymorphic differences in this region of chicken MHC I have been shown to affect peptide selector function when measured *in vitro*, and correlate with changes in protein dynamics^34^. We therefore chose to focus on the α_3_ loop comprising residues 220–227 as a means for tapasin to induce changes in MHC I selector function.

**Figure 5:**
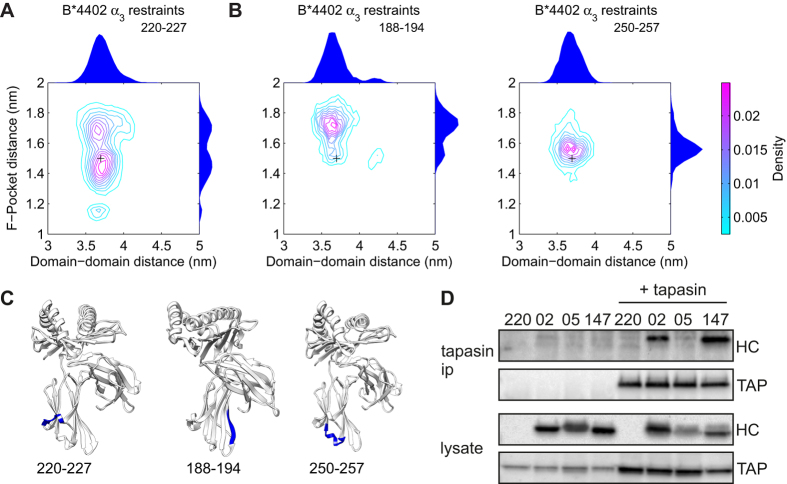
Quantification of B*4402 plasticity with restrained residues at the tapasin binding site, and measurement of tapasin binding for B*4402, B*4405 and B*4405^W147A^. (**A**) Contour plots of the joint probability densities for the conformations populated by peptide free B*4402 with restrained α_3_ domain residues 220–227, the location indicated in panel (**C**). Black crosses indicate the initial structure conformation. Distributions for each individual distance are plotted on the outside of the adjacent axis. Restraint of these residues increases B*4402 plasticity by modulating the peptide binding groove conformation. **(B**) Contour plots of the joint probability densities for the control simulations, the locations are indicated in panel C. Black crosses indicate the initial structure conformation. Distributions for each individual distance are plotted on the outside of the adjacent axis. Restraint of control residues has little effect on B*4402 plasticity. (**C**) Sites of the restraints on MHC I corresponding with simulations in panels A and B. (**D**) B*4405^W147A^ exhibits sustained binding to tapasin, like B*4402, whereas B*4405 does not. Cells were lysed in digitonin to preserve the peptide loading complex, which was then immunoprecipitated with anti-tapasin antibody. Associated transporter associated with antigen processing (TAP) and MHC I (HC) were visualized by Western blot using specific antibodies. See also Figures S1-S5.

Analysis of molecular dynamic simulations (Fig. 3I; Figure S5) revealed a dominant *inter-domain twisting* motion between the peptide binding domain and the α_3_ domain. Furthermore, in peptide-free simulations of B*4402, only the 220–227 loop in the α_3_ domain showed significant motions correlated with F-pocket opening (Fig. 3I). For B*4405, highly correlated motions were observed between the peptide binding domain and the α_3_ domain (Fig. 3H), suggesting a communication between distant regions of MHC I best described as *dynamic coupling* (as illustrated by the covariance webs in Figure S2)^35^,^36^. To further investigate this coupling, we simulated peptide free B*4402 with weak restraints on the C_α_ atoms of residues 220–227 in the α_3_ domain, which also mimics the interaction of tapasin in this region. Figure 5A shows that, under these conditions, the plasticity of the F-pocket of B*4402 was shifted to more closely resemble that of peptide free B*4405, consistent with the existence of dynamic coupling of the α_3_ and peptide binding domains. This effect was a specific property of the 220–227 region, as similar restraints applied to other α_3_ regions (residues 188–194, 250–257) had no significant effect on F-pocket plasticity (Fig. 5B,C).

To determine whether differences in protein plasticity induced by restraints of the α_3_ domain correlated with a physical interaction with tapasin, we measured the binding of tapasin to MHC I *in vivo* for the alleles B*4402, B*4405 and B*4405^W147A^. We performed pull-down experiments to observe co-immunoprecipitation of MHC I with anti-tapasin antibodies from lysates of 220-tapasin-B*4405^W147A^ compared to B*4402 and B*4405. As shown previously, B*4402 had a sustained interaction with tapasin *in vivo*, but B*4405 did not^15^. Strikingly, The W147A mutation conferred sustained tapasin binding to the B*4405 molecule, to a level observed for B*4402 (Fig. 5D). Therefore, we reasoned that B*4405 was able to select high affinity peptides with significantly reduced tapasin interaction because the enhanced plasticity of B*4405 results in enhanced selector function. Whereas B*4402 required tapasin interaction to select high affinity peptides because the reduced plasticity of B*4402 resulted in reduced selector function. By using a restraint to mimic tapasin interaction, we observed B*4402 becoming more plastic. This is consistent with the possibility that tapasin binding to MHC I may be regulated by mobility of the 220–227 loop. Furthermore, the tapasin binding event may be communicated to the peptide binding groove in such a way as to induce plasticity that promotes peptide selection.

### Tapasin enhances peptide selector function by catalyzing transitions between conformational intermediates of MHC I

Finally, we returned to computational systems modelling to infer the mechanisms by which tapasin enhances peptide selector function. We first quantified the effects of tapasin on peptide selection *in vivo*, for the three MHC I alleles B*4402, B*4405 and B*4405^W147A^. As in the absence of tapasin, we developed computational systems models of MHC I peptide selection to encode distinct mechanistic hypotheses, and used Bayesian model selection to determine which hypotheses were most likely, given the experimental data.

**Figure 6:**
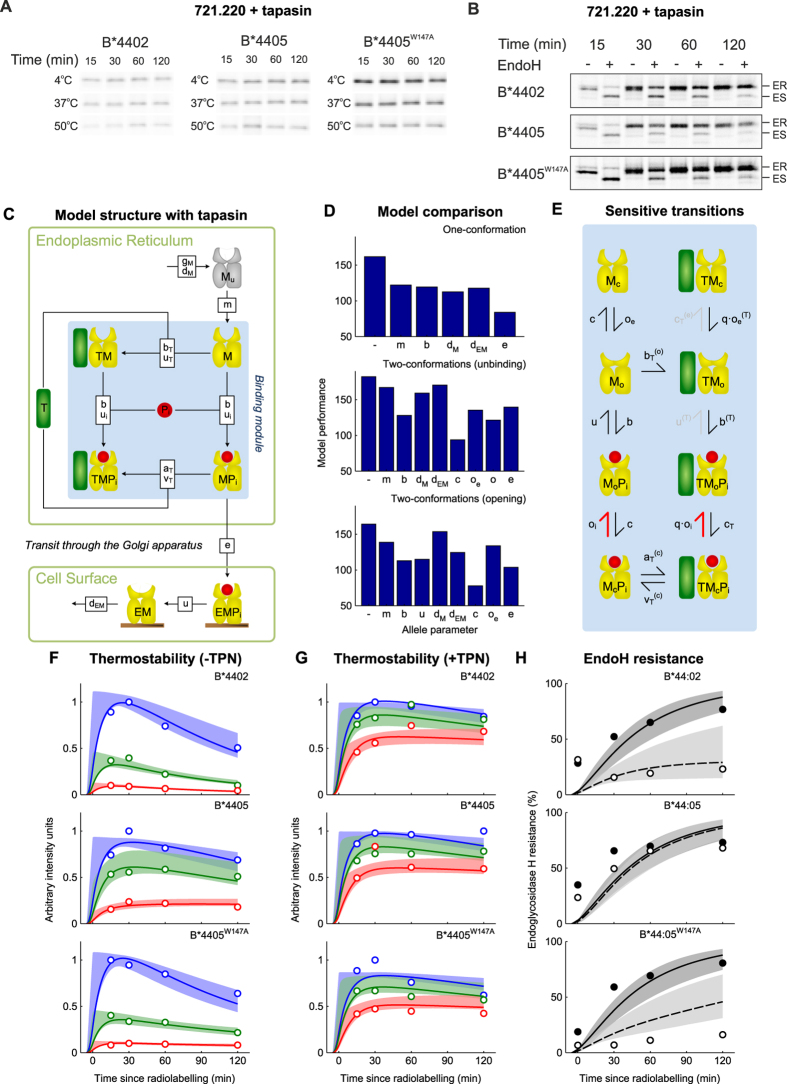
Computational systems models of the mechanisms of MHC I peptide selection, fit to *in vivo* peptide selection data for B*4402, B*4405 and B*4405^W147A^ in the presence and absence of tapasin. (**A**) Repeating the thermostability assay shown in Fig. 2A in the presence of tapasin indicates that B*4402 and B*4405^W147A^ now acquire thermostability equal to that of B*4405 (quantified in G). (**B**) Repeating the pulse chase assay shown in Fig. 2B in the presence of tapasin shows that all alleles select high affinity peptides in the presence of tapasin and traffic to the cell surface (quantified in H). (**C**) Graphical depiction of the *general* computational systems model of the mechanisms of peptide selection in the presence of tapasin, as in Fig. 2D. (**D**) Comparison of model performance for *one-conformation* and *two-conformation* models, as in Fig. 2E. The most likely model (with the lowest BIC score) was once again identified as the two-conformation model with MHC I opening as the peptide-dependent step and MHC I closing rate *c* as the allele-dependent parameter. (**E**) Flux analysis of the two-conformation model with peptide-dependent opening (including tapasin) reveals an anti-clockwise cycle of tapasin mediated peptide editing (the peptide-specific reactions are shown as thick red symbols, and grey lines indicate unfavorable reactions). (**F**–**H**) Comparison of model behavior including a function for tapasin, analogous to Fig. 2F,G. Experimental measurements (circles) quantified from Figs 2A,B and 4A,B and panels (**A**,**B**) in this figure. In **H** solid black are + tapasin and dashed/open are – tapasin.

To quantify peptide selector function in the presence of tapasin, we performed thermostability and EndoH pulse-chase experiments in tapasin-competent cells (Fig. 6A,B), similar to the experiments performed in tapasin-deficient cells (Fig. 2). In the presence of tapasin, all three alleles progressed to the cell surface with the same rapid kinetics (Fig. 6A, quantified in 6G) and loaded rapidly with stabilizing peptides (Fig. 6B, quantified in 6H). This was consistent with the hypothesis that tapasin masked intrinsic differences in MHC I selector function, as previously identified.

To model potential mechanisms by which tapasin could enhance peptide selection, we introduced tapasin into the one-conformation and two-conformation models of Fig. 2, as shown in Fig. 6C (see Supplementary Information and Figure S6 for details of the models). Since tapasin has been shown to elevate loading of stable peptides *in vivo* and to enhance peptide dissociation and association *in vitro*^37^,^38^, for the one-conformation model we assumed that tapasin enhanced peptide binding to and unbinding from MHC I, as previously suggested^27^. For the two-conformation models, we assumed that tapasin enhanced the peptide-dependent step, together with the rates of transition between conformational states.

To determine which mechanistic hypotheses were most likely, we fit the parameters of each model to the experimental data, both in the presence (Figs 2A,B and 4B,C) and absence (Fig. 6A,B) of tapasin, for all three MHC I alleles simultaneously. As in the absence of tapasin (Fig. 2E), the model of best fit was identified as the two-conformation model, with MHC I opening as the peptide-dependent step and MHC I closing rate *c* as the allele-specific parameter (Fig. 6D). Furthermore, as in the absence of tapasin (Fig. 4D), the closing rate for B*4405^W147A^ was identified as lying between the values for B*4402 and B*4405 (Table S2). Consistent with biochemical data^37^,^38^ we observed a close fit of the model to the data when tapasin accelerated both the peptide-dependent rate of opening (factor *q*) and the allele-dependent rate of closing (*c*_*T*_ > *c*) of peptide-bound MHC I (Table S2). Therefore, our modelling results suggest that tapasin confers enhanced selector function by acting as a catalyst to enhance MHC I plasticity, and thus promote peptide exchange.

To understand how specific interactions contribute to the overall behavior of the system, we performed a flux-analysis of the model (Fig. 6E; see Supplementary Information and Figure S6 for details of this analysis). Here, each transition in the model was removed systematically (i.e. set to zero), and the effect on the deviation of the model from the experimental data quantified using the maximum likelihood score. For example, we set the rate of the transition from TM_o_P_i_ to TM_o_ to zero, and found only a small effect on model behavior, which suggests that this transition does not significantly affect peptide selection. This analysis was applied to all transitions and summarized in Fig. 6E, which shows essential transitions left in black, transitions with minor contributions in grey, and non-essential transitions removed. The scheme illustrates some key features of the model: i) open MHC I preferentially binds to tapasin rather than peptide, ii) peptide binds preferentially to tapasin-bound MHC I, and iii) tapasin is predicted to dissociate preferentially from closed, peptide occupied MHC molecules. This is consistent with recent demonstrations that MHC-binding peptides accelerate the dissociation of tapasin from MHC I, measured by surface plasmon resonance^38^.

## Discussion

MHC class I antigen processing can be broadly divided into three phases: peptide generation, peptide selection and peptide presentation. The efficiency of peptide selection in the MHC class I pathway, as well as in the MHC class II pathway^39^, has great relevance for disease pathogenesis^40^,^41^ and therapeutic developments^42^. Hence we sought a mechanism for MHC I peptide selection, by combining modelling and experimental techniques. We highlight three main areas of future investigation. Firstly, here we have simplified a complex system and excluded other factors that may potentially influence peptide selection, such as other chaperone molecules and the nature of the peptide supply^43^. Reducing the system makes the problem tractable in the first instance, and creates a foundation upon which to build more complex systems. Secondly, we note that molecular dynamics simulations in the peptide free state do not have a known corresponding crystal structure: all known structures of MHC I are peptide bound. More generally, our observations from molecular dynamics are dependent upon simulation time and the reliability of the force field to reproduce physical behavior. The simulations presented here are of comparable length, or longer, than those previously performed for these MHC I molecules^22^,^23^,^24^. We anticipate that future NMR studies will test the validity of these observations, however there already exists direct structural evidence for MHC I populating more than one peptide bound conformation^24^,^44^. Thirdly, the differences in MHC I plasticity and corresponding selector function have been shown here for three HLA-B molecules, and the generality of this observation remains to be explored, in particular by considering additional HLA alleles and point mutations. However, there is *in vitro* and computational evidence suggesting that plasticity is a common functional property of MHC I^19^,^20^,^22^,^24^,^45^,^46^ and many other proteins^36^,^47^.

In correlating our observations from the computational systems modelling with those from the molecular dynamics simulations, we can start to build a framework for understanding the mechanisms of MHC I peptide selection. This framework describes how the functional differences in conformational intermediates of distinct MHC I alleles can arise from intrinsic differences in the plasticity of the heavy chain structure encoded by a single amino acid polymorphism. Moreover, we have been able to distinguish between the intrinsic selector properties of MHC I molecules and the impact of the cofactor tapasin in modulating MHC I peptide selection. Our flux analysis identifies that tapasin binds preferentially to conformational intermediates of MHC I and accelerates the closing rate of MHC I, increasing the likelihood of selecting a high affinity peptide. These observations correlate with recent observations of the assembly efficiency of different HLA-B alleles in the absence and presence of tapasin^13^. The correlation with our model is that an MHC I allele with a slow closing rate progresses slowly to the peptide dependent step in the absence of tapasin, thus having a retarded selector function. The model assigns the function of tapasin as one of a catalyst-chaperone, accelerating the rate of closure of MHC I and thus the rate at which MHC I reaches the peptide dependent step. This is consistent with previous investigations indicating that peptide selection by MHC I depends upon conformational change^48^. Our analysis of the flux of MHC I through the model revealed an iterative cycle of tapasin catalyzed peptide exchange, which continues until a peptide of sufficiently high affinity enables MHC I to egress from the ER. This is not dissimilar to the model proposed for HLA-DM and HLA-DR in the MHC Class II pathway^49^,^50^,^51^. From examination of the crystal structure of DR and the DM-DR complex it has been postulated that DM modulates the conformation of peptide free DR such that peptides accessing the peptide binding groove must compete with DM for DR to reverse the conformational change, triggering DM dissociation. This is analogous to tapasin modulated conformational change of MHC I, leading to an increased rate at which MHC I reaches the peptide dependent step in our model, in turn enhancing both the rate, and quantity, of high affinity peptides selected. A key finding in our model is that the difference in intrinsic peptide selection efficiency between alleles can be explained by intrinsic differences in their ability to undergo conformational changes. This is striking because B*4405 and B*4402 differ by only a single amino acid.

Previously, it has been shown by molecular dynamics simulation^23^ that the position 116 polymorphism alters the plasticity of B*4405 and B*4402. By extending these observations to include the B*4405^W147A^ allele we observed a correlation between plasticity and the rate of transition between an open and closed conformation of the peptide binding domain. This suggests a link between plasticity and closing rate such that more plastic MHC I alleles are able to close more quickly. Furthermore, by extending our observations to include the α_3_ domain we can postulate two ways in which plasticity determines peptide selection at a structural level. Firstly, through our observation of coupled dynamics between the membrane proximal α_3_ domain and peptide binding site of the MHC I heavy chain, we see how the 116 polymorphism or the W147A mutation can alter the intrinsic plasticity of these molecules. In the case of B*4405^W147A^ we observe a shift in plasticity towards that of the B*4402 phenotype. Secondly, in demonstrating that we can modulate the conformation of the peptide binding groove of B*4402 via the 220–227 region of the α_3_ domain, we provide an explanation of how tapasin may catalyze peptide selection both directly via the peptide binding site and additionally allosterically: a phenomenon we also recently observed in chicken MHC^34^. It has also been reported that MHC I displays plasticity at the short 3_10_ helix forming part of the peptide-binding groove interacting the N-terminus of bound peptides^20^, and in the α_2-1_ helix of HLA-A*02:01 crystallized with different peptide and T-cell receptor combinations^24^. Further molecular dynamics simulations report how different peptides can influence the plasticity of the MHC I binding groove of HLA-B*27 alleles^22^,^52^, and recent NMR studies report conformational changes in T-cell receptor interaction of H2-L^d^ bound to different peptides^45^. Notably, polymorphisms at position 156 in the α_2-1_ helix in HLA-B*44 alleles, residing at a critical hinge-point important for articulating plasticity have been linked to long-term non-progression in HIV-1 infected individuals^41^, and are associated with HLA-linked drug allergy^40^,^53^ and non-permissive transplantation mismatches^42^. These correlations between protein dynamics and disease pathogenesis are suggestive of the importance of MHC I plasticity throughout the MHC I antigen processing and presentation pathway. Therefore, whilst there remains much to be understood regarding precisely what determines the generation of immunogenic MHC I molecules at the cell surface, the possibility of modulating protein plasticity as a therapeutic target provides an exciting avenue of investigation.

## Methods

### Molecular dynamics simulations

The GROMACS version 4.5.3^30^ molecular dynamics package was used for the all atom simulations with the Amber99SB-ILDN^54^ force field. Further details are provided in the Supplementary Information. Specifically, we present block RMSF calculations in Figure S1 as our check for simulation stability, and also provide a summary of the statistics of the MD simulations in Table S1, including RMSD.

#### Cell lines

The 721.220 cell lines expressing tapasin, B*4402 and B*4405 are previously described^10^. RSV.5neo.B*44:05 was mutated at position 147 by site-directed mutagenesis. Mutation was confirmed by sequencing. 721.220 and 721.220.tapasin cells were transfected using Nucleofection and stable transfectants were grown under G418 and/or puromycin selection. Phenotype was confirmed by FACS and Western blot analysis.

#### BFA Decay

For cell surface decay experiments, cells were treated with Brefeldin A for the specified time points. Cells were stained with W6/32 and analyzed by FACS. Surface MHC I was expressed as percentage of mean channel fluorescence at time point 0.

### Pulse-chase and thermostability assays

These were carried out as previously described^10^ using 10 μCi/ml ^35^S-Translabel and 500 U EndoH. Bands were detected using Personal Molecular Imager FX and quantified using Quantity One software.

### Immunoprecipitations

Cells were lysed in PBS containing 1% Digitonin (WAKO), PMSF and IAA. Supernatants were pre-cleared with protein A sepharose, immunoprecipitated with anti-tapasin PaSta-1 antibody (kind gift from Peter Cresswell) and complexes were recovered with protein A. Beads were washed in 0.1% Digitonin and eluted with Laemmli sample buffer. Eluted proteins and samples of lysate, prior to immunoprecipitation were separated by SDS-PAGE, transferred by Western blot and detected by chemilluminescence using Fluor-S Multimager. Anti-human TAP1, anti-HLA-B (N-20), HRP-conjugated, anti-rabbit light chain and anti-goat antibodies were used.

### Computational modelling

The computational models were constructed as systems of chemical reactions, and simulated as ordinary differential equations assuming mass action kinetics. Numerical integration of the resulting equations and Bayesian parameter inference was performed as described previously^27^. Further details are provided in the Supplementary Information.

## Additional Information

**How to cite this article**: Bailey, A. *et al.* Selector function of MHC I molecules is determined by protein plasticity. *Sci. Rep.* **5**, 14928; doi: 10.1038/srep14928 (2015).

## Supplementary Material

Supplementary Information

## Figures and Tables

**Figure 1:**
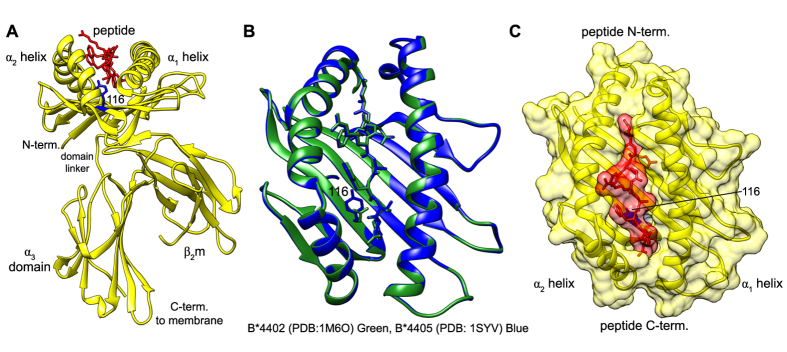
Structure of the MHC I molecule **(A)** Ribbon representation of the MHC I molecule HLA-B*44:05 and its three components: a polymorphic heavy chain (yellow), non-covalently bound invariant β_2_m (yellow) and peptide (red). The polymorphic residue 116 between B*4402 and B*4405 is shown in blue **(B)** Comparison of B*4402 (PDB: 1M6O, green) and B*4405 (PDB: 1SYV, blue) structures. RMSD between them of 0.3 Å. **(C)** Combined ribbon and surface representation of the MHC I molecule peptide binding groove.
