# Supplementary material for: Selector function of MHC I molecules is determined by protein plasticity
Source: Sci Rep. 2015 Oct 20;5:14928. doi: 10.1038/srep14928 (PMC5224517; doi:10.1038/srep14928)
Supplement: Supplementary Information [file srep14928-s1.pdf]

Supplementary Information: Selector function of MHC I molecules is determined by protein plasticity

Alistair Bailey[1,3,4,*], Neil Dalchau[2,*], Rachel Carter[3], Stephen Emmott[2] Andrew Phillips[2], Joern Werner[1,4], and Tim Elliott[1,3]

[1] Institute for Life Sciences, Building 85, University of Southampton, SO17 1BJ, UK
[2] Microsoft Research, 21 Station Road, Cambridge, CB1 2FB, UK
[3] Cancer Sciences Unit, Faculty of Medicine, University of Southampton, Southampton SO16 6YD, UK
[4] Centre for Biological Sciences, Faculty of Natural & Environmental Sciences, Building 85, University of Southampton, SO17 1BJ, UK

[*]These authors contributed equally to this work

Contents

S1 Molecular dynamics simulation of HLA-B*44 molecules **3**
S1.1 Atomistic molecular dynamics simulation protocol . . . 3
S1.2 Simulation stability . . . 5
S1.3 Global motions . . . 6
S1.3.1 Normalised covariance analysis . . . 6
S1.3.2 Functional mode analysis . . . 8
S1.3.3 Probability density analysis . . . 10
S1.3.4 Interpretation of twist angle . . . 11

S2 Kinetic modelling of HLA-B*44 molecules **12**
S2.1 Candidate models . . . 12
S2.1.1 One conformation model . . . 12
S2.1.2 Two-conformations (unbinding) model . . . 13
S2.1.3 Two-conformations (opening) model . . . 17
S2.2 Simulation of thermostability and endoglycosidase H resistance . . . 18
S2.3 Bayesian parameter inference for the kinetic models . . . 18

References **21**

S1 Molecular dynamics simulation of HLA-B*44 molecules

S1.1 Atomistic molecular dynamics simulation protocol

All the molecular dynamic simulations were performed using the IRIDIS High Performance Computing Facility, and we acknowledge the associated support services at the University of Southampton in the completion of this work.

The starting conformations are experimentally determined structures from the RSCB Protein Databank. These were the X-ray crystal structures of HLA-B*44:02, PDB id: 1M6O and HLA-B*44:05, PDB id: 1SYV. The *in silico* W147A point mutation to the structure of HLA-B*44:05 was performed using MODELLER (*1*).

The `GROMACS` version 4.5.3 (*2, 3*) molecular dynamics package was used for the all atom simulations. The simulations used the `Amber99SB-ILDN` (*4*) force field and `TIP3P` (*5*) explicit water molecules using the Simple Point Charge water system (*6*), and Sodium counter ions were added to neutralise the charge of the system. The protein structures were placed in rhombic dodecahedron shaped box centred at 1.5 nm from the edge with periodic boundary conditions. Covalent bond lengths were constrained using the P-LINCS algorithm (*7*) and the water angles were constrained using the SETTLE algorithm (*8*) allowing an integration time step of 2 fs to be used.

Nosé-Hoover temperature coupling (*9, 10*) and Parinello-Rhaman pressure coupling (*11, 12*) used a time constant of 0.5 ps with reference baths of 300 Kelvin and 1 bar respectively to maintain the average thermodynamic properties of the protein and solvent comprising the system. Electrostatic interactions use a cut-off of 1 nm with the interactions beyond this cut-off treated using the particle mesh Ewald method (*13*). Van der Waals forces used a cut-off of 1 nm. The neighbour list is updated every five steps.

Each system initially underwent an energy minimization over 1000 steps of 2 fs to relax the structure and remove the forces from the systems that were introduced by the protonation of the molecule and addition of solvent. This was followed by a 5 ns equilibration of the water surround the protein with the protein atoms restrained using a randomly generated initial starting velocity.

Full production runs were performed with the position restraints released. To analyse conformational dynamics, concatenated trajectories of 420 ns were created from three independent repeats of 150 ns, with the first 10 ns of each simulation discarded. Two additional control simulations were performed over 45 ns each.

For the simulations using distance restraints, a simple force constant of 7.437 kJ mol^{-1} nm^{-2} was applied to the selected C_α atoms for the restraint. This is equivalent to 3 k_bT, where k_b is Boltzmann's constant and T is temperature in Kelvin. For the control simulations we used a strong restraint of 100 kJ mol^{-1} nm^{-2} equivalent to 40 k_bT.

The system components are summarised in Table S1. Parameter files for equilibration and the initial production runs were kindly provided by Tom Piggot (University of Southampton, UK) along with his assistance in the installation and invaluable advice in using `GROMACS`. Quality assurance and post processing was performed using a combination of the suite of utilities provided with `GROMACS`. In some cases these utilities have been adapted as described in the text where relevant. Additional post-processing tasks were performed using MATLAB™ and bespoke UNIX `awk` scripts. Visualisation of the protein structures and molecular dynamics trajectories was performed using the VMD (*14*) and USCF Chimera (*15*) packages.

	System Components			Temperature	RMSD
	Protein atoms	Water	Na^+ Ions	K	nm
HLA-B*44:02 p.b.	6147	26668	12	300	2.58
HLA-B*44:02 p.f.	6002	26717	11	300	2.38
HLA-B*44:05 p.b.	6156	26319	11	300	2.55
HLA-B*44:05 p.f.	6011	26354	10	300	2.44
HLA-B*44:05 W147A p.b.	6142	26014	11	300	2.46
HLA-B*44:05 W147A p.f.	5997	26046	10	300	2.40
HLA-B*44:02 p.f. α_3 rest.	6002	26717	11	300	2.38
HLA-B*44:02 p.f. α_3 rest. cntl.1	6002	26717	11	300	2.37
HLA-B*44:02 p.f. α_3 rest. cntl.2	6002	26717	11	300	2.36

Table S1: Molecular dynamics simulations summary table
Three independent repeats of 150 ns were performed for HLA-B*44:02, HLA-B*44:05 and HLA-B*44:05 W147A. For HLA-B*44:02 p.f. α_3 rest, 5 simulations of 90 ns were performed. For the distance restraint controls 3 simulations of 20 ns were performed. The notation p.b. is peptide bound and p.f. is peptide free. α_3 rest. are distance restrained simulations for positions 220-227. α_3 rest. cntl. are the control distance restrained simulations: 1 are restrained positions 188-194 and 2 are restrained positions 250-257. RMSD is mean value of the backbone atoms versus the average structure from final 130 ns of a representative simulation for the long simulations and the final 10 ns for the short simulations.

S1.2 Simulation stability

The stability and convergence of the molecular dynamics simulations towards an equlibrium state was assessed by calculating the Root Mean Square Fluctuation for blocks of each trajectory as plotted in Figure S1. These indicate that the RMSF between blocks is less than 1 Å and that all the simulations were stable.

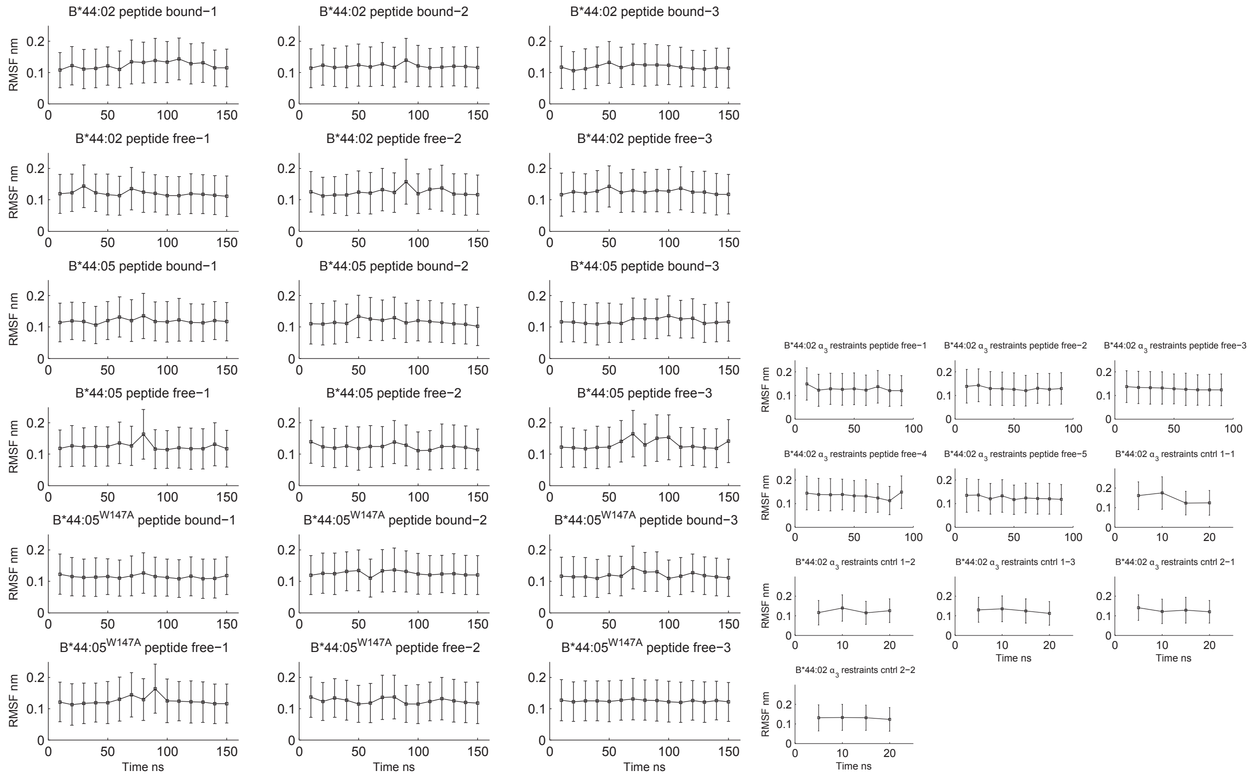

Figure S1: Time block assessment of the stability of the molecular dynamics simulations HLA-B*44:02, HLA-B*44:05, HLA-B*44:05 W147A and HLA-B*44:02 α_3 restrainted . Each plot shows the Root Mean Square Fluctuation (RMSF) of the atoms from their average position during each 10 nanosecond time block of each molecular dynamics simulation trajectory, and for 5 ns time blocks for the short control simulations, as an indication of the overall stability of each simulation and between simulations.

S1.3 Global motions

Global motions of the molecular dynamics simulations were analysed in three ways: Normalised Covariance Analysis, Functional Mode Analysis and Probability Density.

S1.3.1 Normalised covariance analysis

We analysed the degree of correlation between the motions of pairs of atoms of the MHC I complex over the course of the combined simulation trajectory. Highly correlated atoms are moving together and this therefore this indicates they are forming more a rigid body-like structure.
A mass weighted variance-covariance matrix was built using the C_α atoms. This is a symmetric $3N \times 3N$ matrix comprising of the fluctuation of the atom positions with coordinates $\mathbf{x}$ as a function of the trajectory such that:

$$\mathbf{C} = < (\mathbf{x}(\mathrm{t}) - < \mathbf{x} >) \,.\, (\mathbf{x}(\mathrm{t}) - <\mathbf{x}>^{\mathsf{T}}) > \tag{1}$$

where $<>$ indicates the conformational ensemble average. This matrix $\mathbf{C}$ therefore contains as elements, for each atom pair, the difference between the mean product of their atomic positions and the product of their mean atom positions *i.e.* the difference between their average position as a pair and the product of their individual average positions. Atom pairs moving together in the same direction give rise to positive covariances and pairs moving in the opposite direction give rise to negative covariances. Non-correlated atoms give near zero covariances. The variance for each atom is contained on the main diagonal.

We calculate the normalized covariance of the atoms by summing the x, y and z components of the matrix and normalizing with the self-covariance of the atoms. Re-expressing equation 1 for atoms i and j as:

$$\mathbf{C}_{\mathrm{ij}} = < (\mathbf{x}_{\mathrm{i}}(\mathrm{t}) - < \mathbf{x}_{\mathrm{i}} >) \,.\, (\mathbf{x}_{\mathrm{j}}(\mathrm{t}) - <\mathbf{x}_{\mathrm{j}} >> \tag{2}$$

the normalized covariance is:

$$\mathbf{C}'_{\mathrm{ij}} = \frac{\mathbf{C}_{x_i x_j} + \mathbf{C}_{y_i y_j} + \mathbf{C}_{z_i z_j}}{\sqrt{(\mathbf{C}_{x_i x_i} + \mathbf{C}_{y_i y_i} + \mathbf{C}_{z_i z_i}).(\mathbf{C}_{x_j x_j} + \mathbf{C}_{y_j y_j} + \mathbf{C}_{z_j z_j})}} \tag{3}$$

This yields a matrix containing the correlations between the atoms in terms of the Pearson Correlation Coefficient for each pair of atoms. Extracting atom pairs above a given magnitude of correlation *e.g.* greater than 0.7, it is possible to create an image of the correlated atoms on the three dimensional structure as a web of connections (*16*). This gives an indication of which atoms are moving together during the simulation which in turn indicates parts of the structure that are acting as rigid bodies or where there is potential communication between parts of the structure. It does not however indicate the magnitude of the motions, only that there are correlated above a certain threshold *i.e.* there is a linear relationship between the motion of correlated atoms as defined by the strength of correlation coefficient chosen. Therefore this analysis cannot tell us anything about any non-linear relationships between the atoms of the protein. For this analysis we used an amended version of the `GROMACS` `g_covar` utility and the `g_anaeig` utility.

We used a common peptide free reference structure with the combined trajectories for the analysis. This therefore excludes correlations with the peptide in the peptide bound state.
Fig. S2 shows the web of correlations between all pairs of C_α atoms with a normalised covariance of greater than 0.7 for the peptide bound and peptide free HLA-B44 simulations. It's important to reiterate this is simply a dimensionless number indicating the degree above which we observe a correlation between a pair of atoms and that the choice of 0.7 is some what arbitrary. The choice of C_α atoms is simply to provide clarity in visualization of the correlations.

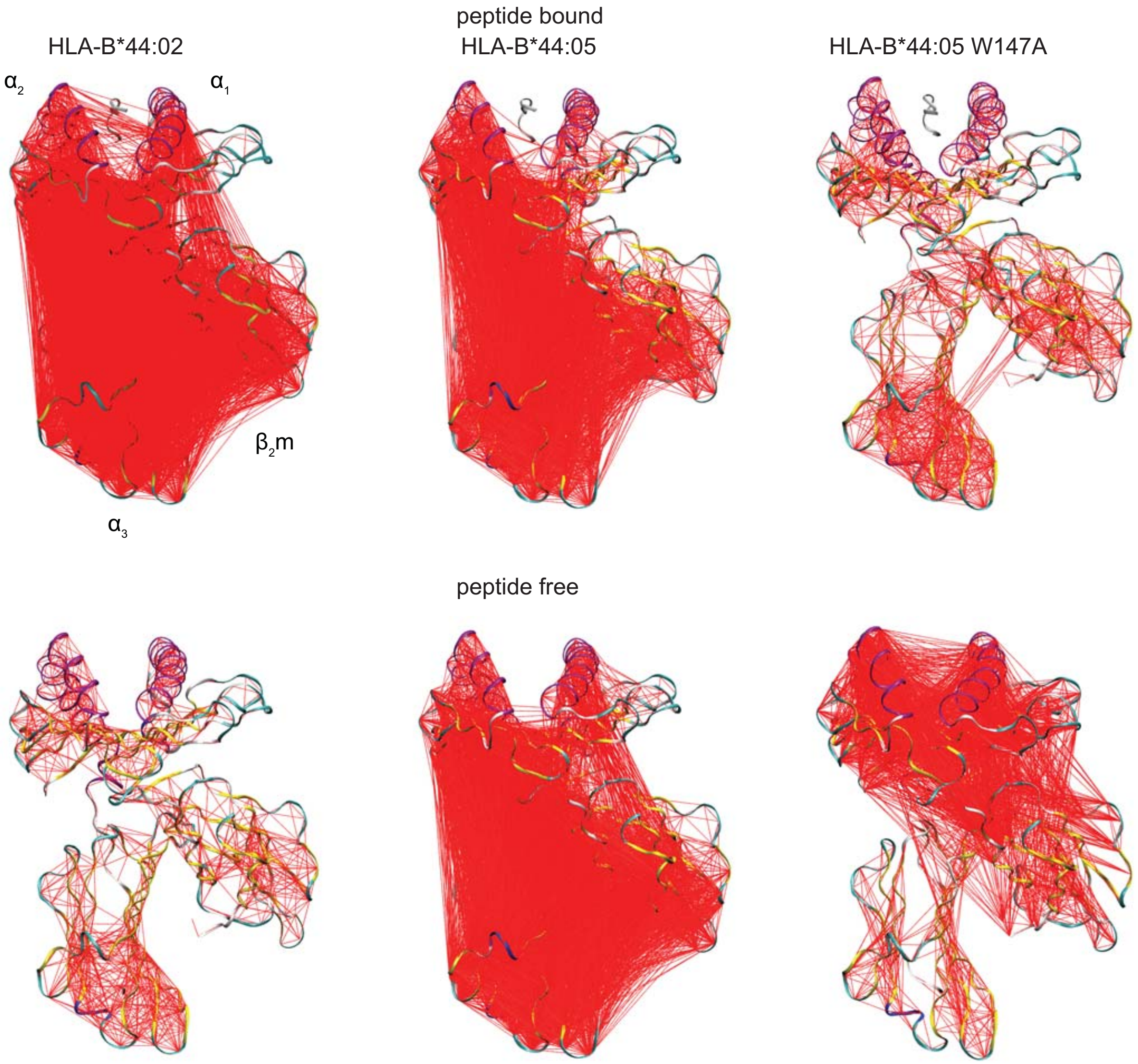

Figure S2: HLA-B*44 Normalised covariance webs
C_α atom pairs with a covariance yielding a correlation coefficient greater than 0.7 are shown connected by red lines for the peptide bound and peptide free simulations. The red lines indicate that connected atoms move together during the simulation, but not the direction or magnitude of the motion. Correlations with the peptide have been excluded for clarity. The calculations were done using an amended version of the `GROMACS` `g_covar` utility and the `g_anaeig` utility.

S1.3.2 Functional mode analysis

Having performed Principal Component Analysis using the GROMACS g_covar and the g_anaeig utilities, as previously described (*17*), to extract top 50 modes accounting for ∼90% of the total atomic motion, we performed Functional Mode Analysis (FMA) of peptide free MHC, as proposed and implemented by Jochen Hub and Bert de Groot, . It aims to detect the collective atomic motions directly connected to protein function. It therefore requires the selection of a "functional quantity" that describes the functional state of the protein to be correlated with the protein motion. A full description of the theory and the method is detailed in the publication by Hub (*18*).

In the absence of experimental data to guide the choice of a functional quality, we make the observation that in the X-ray crystal structure the peptide is buried inside the peptide binding groove with no easy means of entry or exit. Yet we know that peptides load and can be exchanged. We identified a potential hinge in the α_{2-1} helix and measured the distance fluctuations in this region to define a functional quantity for peptide binding and unbinding. It only makes sense to consider this for peptide free MHC as in the peptide bound state there are no significant distance fluctuations.

Correlating the changes in distance with the overall motion of the molecule as described by the principal components yields a single collective motion for MHC I associated with the conformational changes in the peptide binding groove. The method is implemented using the common reference structure created for principal component analysis and the FMA package developed by Jochen Hub (*18*):

1. From identification of the two hinge points common in the α_{2-1} helix, two regions were defined on either side of the F-pocket of the MHC molecule peptide binding groove. Residues 135 to 156 on the α_{2-1} helix and residues 69 to 85 on α_1 helix as coloured red in Fig. S3

2. For each combined trajectory in the peptide bound and peptide free states the distance was measured between the centres of mass of these two regions over the duration of the concatenated simulation. This distance is a function of time $d_{\alpha_1\alpha_2}$ and is the functional quantity.

3. Assuming $d_{\alpha_1\alpha_2}$ is approximately a linear function of the principal components, a collective vector **a** was constructed from the principal components derived by diagonalization of the covariance matrix. As the first 50 principal components account for ∼90% of the atomic mean square atomic fluctuations, this subset of the principal components was considered a reasonable set for the construction of **a**.

4. Quantifying the correlation using the Pearson correlation coefficient R, the motion along **a** is maximally correlated to the change in $d_{\alpha_1\alpha_2}$ to yield the Maximally Correlated Motion (MCM) as function of time given by projection p_a, such that:

$$R = \frac{\mathrm{cov}\,(d_{\alpha_1\alpha_2}, \mathrm{p}_a)}{\sigma_{d_{\alpha_1\alpha_2}}.\sigma_a} \tag{4}$$

5. The process of maximizing the R generates a model for $d_{\alpha_1\alpha_2}$ as a function of the principal components. The model is cross validated (Fig. S3) by dividing the trajectory into model building frames and cross validation frames. We used 350 ns for model building and 70 ns for cross validation. We test the ability of the model to predict the value of R in the cross validation set as compared with that calculated from the data (Fig. S3). This was analysed to determine contributions from the different principal components to the variance of the model and therefore the influence of individual principal components on $d_{\alpha_1\alpha_2}$ (Fig. S3)

6. The MCM was then further optimized to find the most probable motion given the input ensemble of structures. This yields the ensemble-weighted Maximally Correlated Motion (ewMCM) in accordance with the free energy landscape described by the input trajectory (*18*). This estimates the most probable collective motion for the MHC Class I molecule that achieves a substantial change in $d_{\alpha_1\alpha_2}$ which is visualised as a projection of the trajectory onto this eigenvector (Fig. S3).

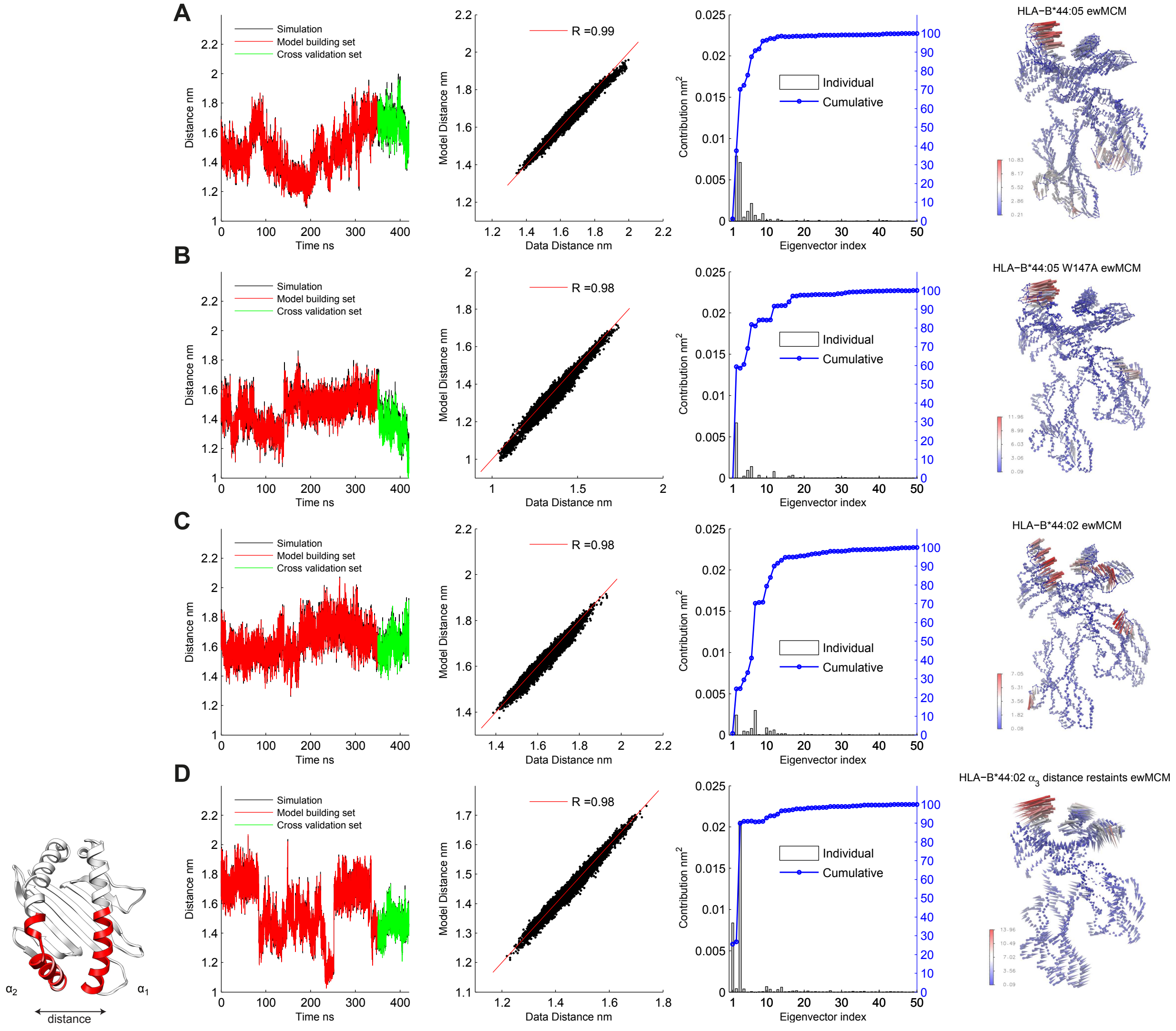

Figure S3: HLA-B*44 peptide free functional mode analysis
Using two hinge points in the α_{2-1} helix, two regions were defined on either side of the F-pocket of the MHC molecule peptide binding groove. Residues 135 to 156 on the α_{2-1} helix and residues 69 to 85 on α_1 helixwo regions were defined on either side of the F-pocket of the MHC molecule peptide binding groove. as coloured red and the distance between the centres of mass of these backbone atoms was measured as a function of simulation time to become functional quantity $d_{\alpha_1\alpha_2}$. For each peptide free HLA-B*44 molecule, panels **A-D**, a model building set of 350 ns is shown in red and the resulting cross-validation model prediction for the $d_{\alpha_1\alpha_2}$ shown in green over the actual measurements in black. A scatter plot of the data versus the cross validation set predictions. This plot indicates the individual contributions of each eigenvector used in constructing the model to the variance in $d_{\alpha_1\alpha_2}$ in grey bars and the cumulative contribution in blue. The resulting ensemble weighted Most Correlated Motion contributing to a change in the distance across the F-pocket shown as a porcupine plot. Cones attached to each backbone atom indicate the direction and amplitude of motion of this mode in Å.

S1.3.3 Probability density analysis

Probability densities were calculated to characterise the conformations explored by the MHC structures in two ways, one using intra-molecular distances and the other combining intra-molecular distance and angle between the heavy chain domains. Intra-molecular distances were chosen from the identification of the two common hinge points in the α_2 helix from the conformational angles analysis, two regions are defined on either side of the F-pocket of the MHC molecule peptide binding groove. Residues 135 to 156 on the α_2 helix and residues 69 to 85 on α_1 helix as coloured red in Fig. S4A. The domain-domain distance was defined as between residues 96-100 in the peptide binding groove platform and the flexible α_3 domain region 220-227, also coloured red in Fig. S4A. These distances were measured for the combined simulations using the `GROMACS` utility `g_dist` and joint probability densities for these two distances were then calculated using MATLAB™ as plotted in Figures 4 and 5 in the main manuscript.

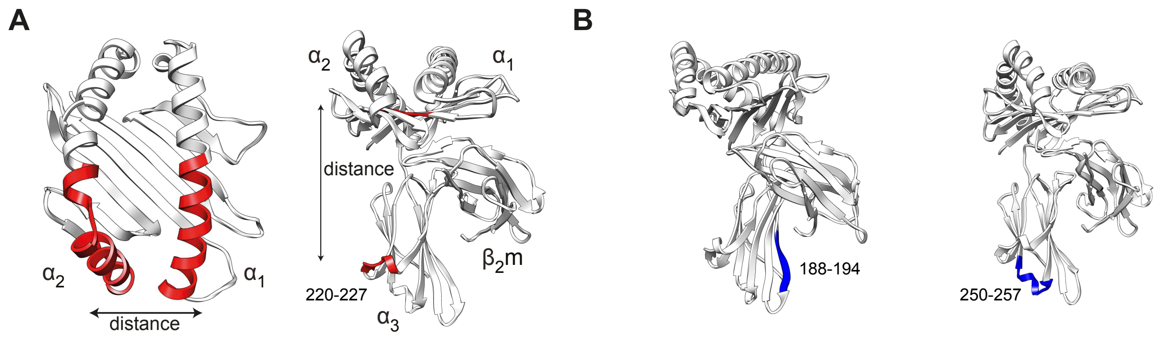

Figure S4: HLA-B*44 joint distance probability densities
A. Two distances were measured between the centre of mass of backbone atoms of the regions coloured red. The distance across the F-pocket was defined as between residues 135 to 156 on the α_2 helix and residues 69 to 85 on α_1 helix. The domain-domain distance was defined as between residues 96-100 in the peptide binding groove platform and the loop 220-227 in the α_3 domain. The distances were calculated using the `GROMACS` utility `g_dist`. Residues 220-227 were used for the peptide free restrained simulations of HLA-B*44:02. **B.** Coloured blue on the structure of MHC I are the sites at which the residues were restrained, 188-194 and 250-257, for the restrained control simulations of HLA-B*44:02 in the peptide free state.

S1.3.4 Interpretation of twist angle

For the angle of twist between peptide binding domain and the α_3 two planes represented as discs in Fig. S5A were defined through the centre of mass of the heavy chain by the C_α atoms of residues 118, 174 and 252 and the centre of mass of the α_3 domain by residues 199, 209 and 260. The angle between the normal to these planes θ during the 420 ns combined simulations measures the twisting angle in degrees. This angle was calculated using `GROMACS` utility `g_sgangle`. The range of this twisting angle for each HLA-B*44 molecule is shown in Figure 1F of the main text. Joint probability densities for the F-pocket distance defined in Fig. S4A and the domain-domain twisting were then calculated using MATLAB™ as plotted in Fig. S5C-E.

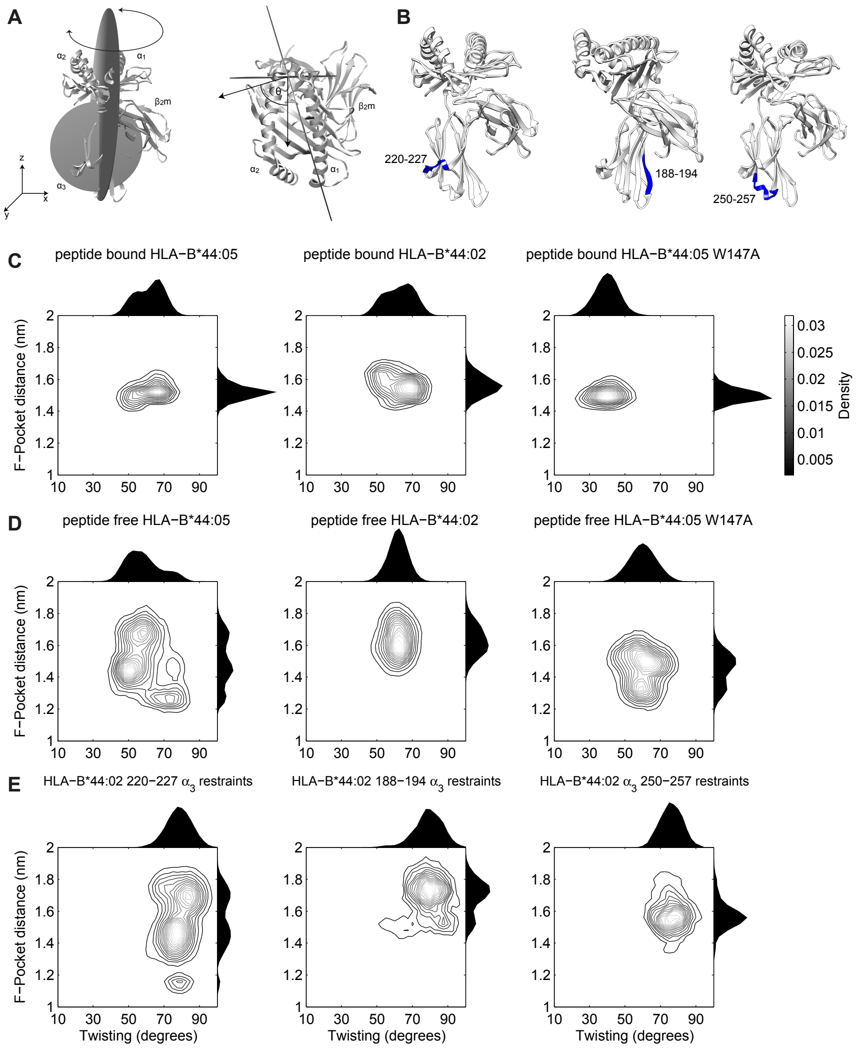

Figure S5: HLA-B*44 joint twist probability densities
A. The twist angle was defined by two planes represented as discs here were defined through the centre of mass of the heavy chain by the C_α atoms of residues 118, 174 and 252 and the centre of mass of the α_3 domain by residues 199, 209 and 260. The angle between the normal to these planes θ during the 420 ns combined simulations measures the twisting angle between the peptide binding domain & the α_3 domain. The angle was calculated using `GROMACS` utility `g_sgangle`. **B.** Coloured blue on the structure of MHC I are the restrained regions indicated in blue were 220-227 and controls of 188-194 and 250-257. **C-E.** The MATLAB™ `hist3` utility was used to create a bivariate histogram from which the probability density function is calculated and plotted for the distance change across the F-pocket of the peptide binding groove against the twisting angle between the peptide binding domain & α_3 domain.

S2 Kinetic modelling of HLA-B*44 molecules

S2.1 Candidate models

S2.1.1 One conformation model

Previously, we constructed a model of MHC class I that could describe the time-dependent optimisation of peptide cargo (*19*). Emanating from this work was the prediction that HLA-B molecules vary in their intrinsic ability to load high affinity peptides, which influences the extent to which tapasin confers an additional optimisation benefit via skewing the competition between tapasin binding and peptide binding.

In this study, we sought to determine whether this model could be extended to incorporate a conformational intermediate, and understand more specifically how peptide loading might be altered in different HLA-B molecules. Before defining the two conformations models, we introduce the original one-conformation model, detailing some alterations that were made and propagated to the two-conformations models.

1. As peptide supply and turnover is much faster than the other kinetics in the system, we assume that the concentration of peptide is in equilibrium. i.e. $[P_i] \equiv P_i$.

2. After collecting temporally resolved data for the three HLA-B molecules investigated herein, we noticed that the abundance of recoverable radioactive molecules sometimes increased between the data-points at 15 minutes and 30 minutes after the termination of radio-labelling. Therefore, on this timescale, some process must occur that changes MHC I molecules from an immature state to a mature state (in terms of its recognition by antibody). This could be the effect of $\beta_2 m$ binding, calnexin unbinding, both, or neither. To account for the effect (which could be explored in detail in another study), we modelled this as a first order reaction in which unrecoverable molecules (M_u) transition into mature molecules (M) with rate m, following endoplasmic reticulum (ER)entry of immature MHC I molecules at rate g_M.

3. Changing the v_T reaction to reversible, with rate a_T,

Applying these two alterations to the original model gives rise to

$$\begin{array}{ccccccccc}
\varnothing & \underset{d_T}{\overset{g_T}{\rightleftharpoons}} & T & M & \underset{u_i}{\overset{b\cdot P_i}{\rightleftharpoons}} & MP_i & MP_i & \xrightarrow{e} & EMP_i \\
\varnothing & \underset{d_M}{\overset{g_M}{\rightleftharpoons}} & M & TM & \underset{q\cdot u_i}{\overset{a\cdot P_i}{\rightleftharpoons}} & TMP_i & EMP_i & \xrightarrow{u_i} & EM \\
T+M & \underset{u_T}{\overset{b_T}{\rightleftharpoons}} & TM & T+MP_i & \underset{v_T\ T}{\overset{a}{\rightleftharpoons}} & TMP_i & EM & \xrightarrow{d_{EM}} & \varnothing
\end{array}$$

Assuming mass action, the corresponding ODEs are given by

$$\frac{d[M_u]}{dt} = g_M - [M_u](d_M + m) \tag{5a}$$

$$\frac{d[M]}{dt} = m[M_u] + \sum_i u_i[MP_i] + u_T[TM] - (b\sum_i P_i + b_T[T] + d_M)[M] \tag{5b}$$

$$\frac{d[T]}{dt} = g_T + u_T[TM] + v_T\sum_i[TMP_i] - (b_T[M] + a_T[MP_i] + d_T)[T] \tag{5c}$$

$$\frac{d[TM]}{dt} = b_T[M][T] + q\sum_i u_i[TMP_i] - (u_T + a\sum_i P_i)[TM] \tag{5d}$$

$$\frac{d[MP_i]}{dt} = b\cdot P_i[M] + v_T[TMP_i] - (u_i + a_T[T] + e)[MP_i] \tag{5e}$$

$$\frac{d[TMP_i]}{dt} = a\cdot P_i[TM] + a_T[T][MP_i] - (qu_i + v_T)[TMP_i] \tag{5f}$$

$$\frac{d[EMP_i]}{dt} = e[MP_i] - u_i[MeP_i] \tag{5g}$$

$$\frac{d[EM]}{dt} = \sum_i u_i[MeP_i] - d_{EM}[Me] \tag{5h}$$

Solving the system at steady state, with $a_T = 0$, leads to the following expression for the populations of egressed peptide-MHC I complexes, as previously derived (*19*)

$$[EMP_i]^* = \frac{1}{u_i}\frac{e}{u_i + e}(b[M]^* + \frac{x}{u_i + x}a[TM]^*)P_i \tag{6}$$

S2.1.2 Two-conformations (unbinding) model

To incorporate the possibility of a conformational change that alters peptide binding and unbinding, we first proposed a model that extends the one-conformation model simply by using two conformational states for all MHC I molecules (Fig. 1B and S6). These are distinguished as molecules that are able to load peptide ("open") and those that are not able to load peptide (i.e. "closed"). Transitions between open and closed states proceed by opening rates o and o_e for peptide-bound and empty molecules respectively, and closing rates c and c_T for tapasin-free and tapasin-bound molecules respectively. Additionally, we assumed that MHC I molecules transition to the closed state (M_c) immediately following *maturation* from the state M_u. As we explicitly model a maturation process during which time no peptide binding can occur, we felt that this assumption would lead to very minor differences to the alternative assumption of $M_u \rightarrow M_o$. As for the one-conformation model, we allowed tapasin to enhance peptide dissociation by a factor q.

The underlying reactions for the two-conformations (unbinding) model are given by (see Fig. S6a for a graphical depiction)

$$\begin{array}{rcl@{\qquad}rcl@{\qquad}rcl}
\varnothing & \underset{d_T}{\overset{g_T}{\rightleftharpoons}} & T & \varnothing & \underset{d_M}{\overset{g_M}{\rightleftharpoons}} & M_u & M_cP_i & \overset{e}{\rightarrow} & EM_cP_i \\
M_u & \overset{m}{\rightleftharpoons} & M_c & M_c & \underset{o_e}{\overset{c}{\rightleftharpoons}} & M_o & M_oP_i & \overset{e}{\rightarrow} & EM_oP_i \\
M_o, M_c & \overset{d_M}{\longrightarrow} & \varnothing & T + M_c & \underset{u_T}{\overset{b_T}{\rightleftharpoons}} & TM_c & EM_cP_i & \underset{o}{\overset{c}{\rightleftharpoons}} & EM_oP_i \\
T + M_o & \underset{u_T}{\overset{b_T}{\rightleftharpoons}} & TM_o & TM_c & \underset{o_e}{\overset{c_T}{\rightleftharpoons}} & TM_o & EM_oP_i & \overset{u_i}{\rightarrow} & EM_o \\
M_o & \underset{u_i}{\overset{b \cdot P_i}{\rightleftharpoons}} & M_oP_i & TM_o & \underset{q.u_i}{\overset{b \cdot P_i}{\rightleftharpoons}} & TM_oP_i & EM_o & \overset{d_{EM}}{\longrightarrow} & \varnothing \\
M_oP_i & \underset{c}{\overset{o}{\rightleftharpoons}} & M_cP_i & TM_oP_i & \underset{c_T}{\overset{o}{\rightleftharpoons}} & TM_cP_i & & & \\
T + M_oP_i & \underset{v_T\ T}{\overset{a}{\rightleftharpoons}} & TM_oP_i & T + M_cP_i & \underset{v_T\ T}{\overset{a}{\rightleftharpoons}} & TM_cP_i & & &
\end{array}$$

where the egression of open peptide-bound MHC I molecules (M_oP_i) is coloured red to indicate its inclusion is to be decided upon. Assuming mass action kinetics, we can write down the corresponding ODEs as

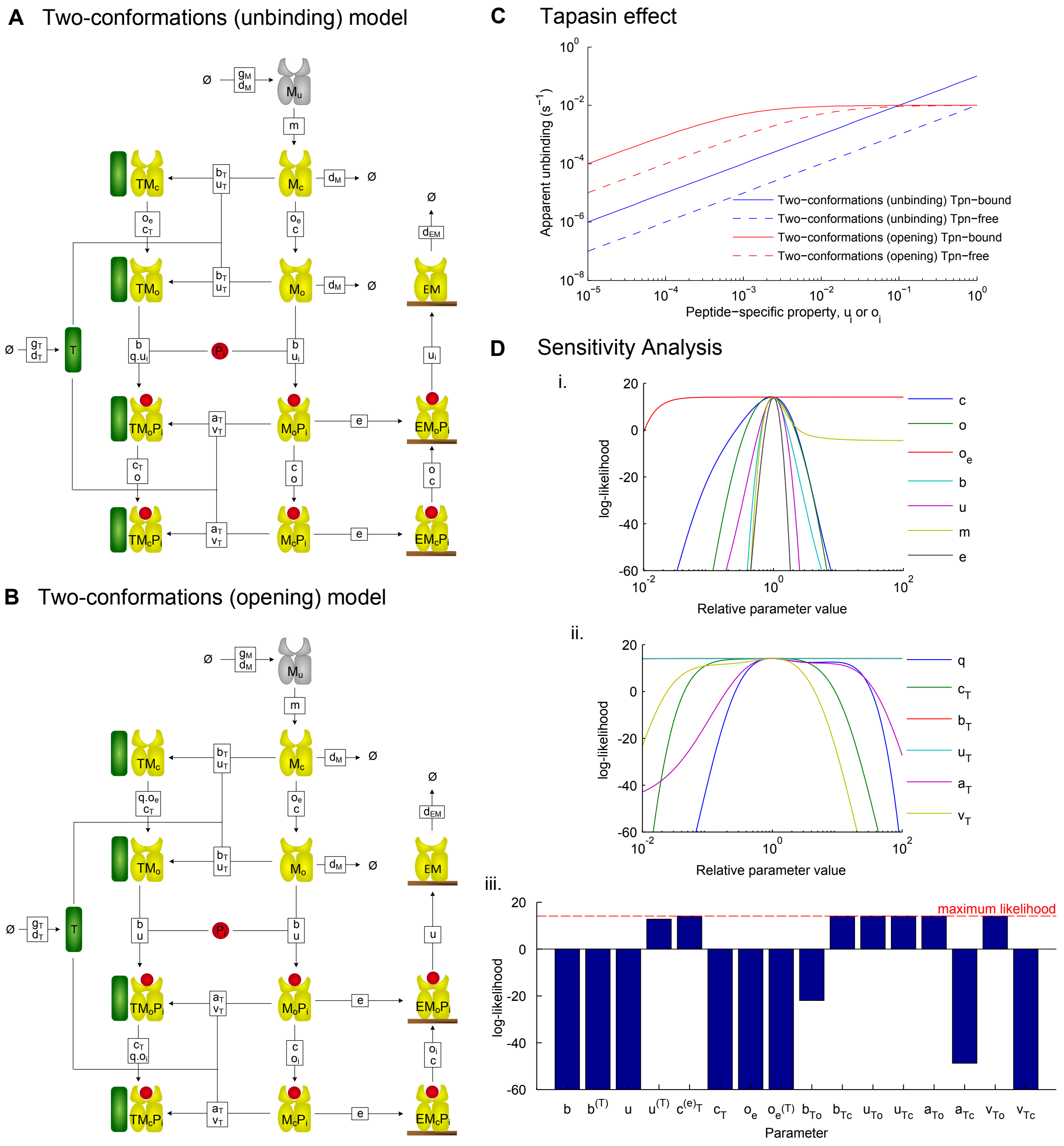

Figure S6: Two-conformation models of MHC class I peptide loading. **(A,B)** Graphical depiction of two-conformations models, where (A) the unbinding rate of M_oP_i, or (B) the opening rate of M_cP_i, is the peptide-dependent reaction. Each shape in the model represents a molecular species and each box represents a reaction, where inbound edges represent reactants and outbound edges represent products. Boxes are labelled with corresponding reaction rates, where a single rate denotes an irreversible reaction and two rates denote a reversible reaction, with the rate of the forward reaction indicated on top. **(C)** The theoretical effect of tapasin on peptide dissociation is plotted, based on the analysis in Section S2.1. For the two-conformations (unbinding) model, the apparent dissociation was calculated over a range of u_i according to equations 8c and 8d with $c = 10^{-2}$, $c_T = 1$, $o = 10^{-4}$ and $q = 1000$. For the two-conformations (opening) model, the apparent dissociation was calculated over a range of o_i according to equations 10c and 10d with $c = 10^{-2}$, $c_T = 1$, $u = 10^{-2}$ and $q = 1000$. **(D)** Sensitivity analysis of the two-conformations (opening) model with allele-specific closing. The log-likelihood was computed for parametric deviations from the maximum likelihood parameter set. In each case, a single parameter was varied over 4 orders of magnitude. The parameters analyzed are grouped as (i) non-tapasin-associated, and (ii) tapasin-associated. As the parameters c and o are multi-valued (allele-specific and peptide-specific respectively), the results are indicative of changing all values equivalently. (iii) The flux through different pathways in the model was analyzed by setting equal to zero the parameters of tapasin binding to and unbinding from MHC/pMHC molecules.

$$\frac{d[M_u]}{dt} = g_M - [M_u]\left(d_M + m\right) \tag{7a}$$

$$\frac{d[M_c]}{dt} = m[M_u] + c[M_o] + u_T[TM_c] - [M_c]\left(d_M + o_e + b_T[T]\right) \tag{7b}$$

$$\frac{d[M_o]}{dt} = u_T[TM_o] + \sum_i u_i[M_oP_i] + o_e[M_c] - [M_o]\left(d_M + b\sum_i[P_i] + c + b_T[T]\right) \tag{7c}$$

$$\begin{aligned}\frac{d[T]}{dt} &= g_T + u_T[TM_c] + u_T[TM_o] + v_T\sum_i[TM_oP_i] + v_T\sum_i[TM_cP_i]\ldots \\ &- [T]\left(d_T + b_T[M_c] + b_T[M_o] + a_T\sum_i[M_oP_i] + a_T\sum_i[M_cP_i]\right)\end{aligned} \tag{7d}$$

$$\frac{d[TM_c]}{dt} = b_T[T][M_c] + c_T[TM_o] - [TM_c]\left(u_T + q\cdot o_e\right) \tag{7e}$$

$$\frac{d[TM_o]}{dt} = q\cdot o_e[TM_c] + b_T[T][M_o] + q\sum_i u_i[TM_oP_i] - [TM_o]\left(c_T + u_T + b\sum_i[P_i]\right) \tag{7f}$$

$$\frac{d[M_cP_i]}{dt} = c[M_oP_i] + v_T[TM_cP_i] - [M_cP_i]\left(o + a_T[T] + e\right) \tag{7g}$$

$$\frac{d[M_oP_i]}{dt} = b[M_o][P_i] + o[M_cP_i] + v_T[TM_oP_i] - [M_oP_i]\left(u_i + c + a_T[T] + e\right) \tag{7h}$$

$$\frac{d[TM_cP_i]}{dt} = c_T[TM_oP_i] + a_T[T][M_cP_i] - [TM_cP_i]\left(o + v_T\right) \tag{7i}$$

$$\frac{d[TM_oP_i]}{dt} = b[TM_o][P_i] + o[TM_cP_i] + a_T[T][M_oP_i] - [TM_oP_i]\left(q\cdot u_i + c_T + v_T\right) \tag{7j}$$

$$\frac{d[EM_cP_i]}{dt} = e[M_cP_i] + c[EM_oP_i] - o[EM_cP_i] \tag{7k}$$

$$\frac{d[EM_oP_i]}{dt} = e[M_oP_i] + o[EM_cP_i] - [EM_oP_i]\left(c + u_i\right) \tag{7l}$$

$$\frac{d[EM_o]}{dt} = \sum_i u_i[EM_oP_i] - d_{EM}[EM_o] \tag{7m}$$

Relationship to the one-conformation model

In order to determine the consequences of the additional conformational state, we derive the one-conformation model from these equations. Intuition suggests that the one-conformation model is approximately equivalent to the *closing* and *opening* rates being infinitely fast. Therefore, we apply the following substitutions:

$$[M_c] = \frac{c}{o_e}[M_o]$$

$$[TM_c] = \frac{c_T}{o_e}[TM_o]$$

$$[M_cP_i] = \frac{c}{o}[M_oP_i]$$

$$[TM_cP_i] = \frac{c_T}{o}[TM_oP_i]$$

By also defining $[M] = [M_c] + [M_o] = \frac{c+o_e}{c}[M_o]$, $[TM] = [TM_c] + [TM_o] = \frac{c_T+o_e}{c_T}[TM_o]$, etc., the system can be written as

$$\begin{aligned}
\frac{d[T]}{dt} &= g_T + u_T[TM] + v_T \sum_i [TMP_i] + -[T]\left(d_T + b_T[M] + a_T \sum_i [MP_i]\right) \\
\frac{d[M_u]}{dt} &= g_M - [M_u]\left(d_M + m\right) \\
\frac{d[M]}{dt} &= m[M_u] + u_T[TM] + \frac{o}{c+o}\sum_i u_i[MP_i] - [M]\left(d_M + b_T[T] + \frac{o_e}{c+o_e}\cdot b \sum_i P_i\right) \\
\frac{d[TM]}{dt} &= b_T[T][M] + \frac{o}{c_T+o} q \sum_i u_i[TMP_i] - [TM]\left(u_T + \frac{o_e}{c_T+o_e}\cdot b\sum_i P_i\right) \\
\frac{d[MP_i]}{dt} &= \frac{o_e}{c+o_e}\cdot b[M]P_i + v_T[TMP_i] - [MP_i]\left(\frac{o}{c+o}\cdot u_i + a_T[T] + e\right) \\
\frac{d[TMP_i]}{dt} &= \frac{o_e}{c_T+o_e}\cdot b[TM]P_i + a_T[T][MP_i] - [TMP_i]\left(\frac{o}{c_T+o}\cdot q\cdot u_i + v_T\right) \\
\frac{d[EMP_i]}{dt} &= e[MP_i] - \frac{o}{c+o}\cdot u_i[EM_oP_i] \\
\frac{d[EM]}{dt} &= \frac{o}{c+o}\cdot \sum_i u_i[EM_oP_i] - d_{EM}[EM]
\end{aligned}$$

which is precisely equivalent to the one-conformation model with

$$b \to \frac{o_e}{c+o_e}\cdot b = \hat{b} \tag{8a}$$

$$a \to \frac{o_e}{c_T+o_e}\cdot b = \hat{a} \tag{8b}$$

$$u_i \to \frac{o}{c+o}\cdot u_i \tag{8c}$$

$$q\cdot u_i \to \frac{o}{c_T+o}\cdot q\cdot u_i = \hat{q}\cdot u_i \tag{8d}$$

By observing these relationships, we can interpret the tapasin enhancement of the peptide on-rate as

$$a_r = \frac{\hat{a}}{\hat{b}} = \frac{c+o_e}{c_T+o_e}$$

and the Tapasin enhancement of the peptide off-rate as

$$\hat{q} = \frac{c+o}{c_T+o}\cdot q$$

This enables us to re-interpret the filter principle in the two-conformations model, assuming that closing and opening occur on a faster timescale than peptide binding/unbinding (note that for comparison with the one-conformation model, we assume $a_T = 0$ in the following). This gives

$$\begin{aligned}
[EMP_i]^* &= \frac{b}{u_i}\cdot\frac{e}{\frac{o}{c+o}\cdot u_i + e}\left(\frac{o_e}{c+o_e}\cdot[M]^* + \frac{o_e}{c_T+o_e}\cdot\frac{v_T}{\frac{o}{c_T+o}\cdot q\cdot u_i + v_T}\cdot[TM]^*\right)\cdot P_i \\
&\propto \frac{1}{u_i}\cdot\frac{e'}{u_i+e'}\cdot\left([M]^* + a_r\cdot\frac{x}{x+u_i}\cdot[TM]^*\right)[P_i]^*
\end{aligned} \tag{9}$$

where $e' = e\cdot\frac{c+o}{o}$ and $x = \frac{v_T}{q}\cdot\frac{c_T+o}{o}$. Note that this equation has the same form as for the one-conformation model (6), so the filtering relation is unchanged for the two-conformations (unbinding) model.

S2.1.3 Two-conformations (opening) model

Based on an argument related to thermodynamics, it is more likely that the peptide-dependent step of peptide-MHC I unbinding is the rate at which a "closed" molecule becomes "open". i.e. rate o in the two-conformations model. This argument rests on the observation that the extent of the interactions a peptide makes with a MHC I molecule would be greater in a closed state than an open MHC I state and therefore the number and quality of these interactions would determine the opening rate of MHC I in a peptide-dependent manner. Therefore, we propose a variation to the two-conformations model that is as close as possible to the other models, except that the rate of opening of a peptide-bound MHC I molecule is peptide-dependent, while the rate of peptide-MHC I disassociation is homogeneously assigned. We propose that the effects of tapasin are to increase peptide unloading by increasing the peptide-dependent opening rate ($o_i \to q \cdot o_i$), and to modify the rate of closing ($c \to c_T$), though this time having no effect on peptide binding (rate b) and unbinding (rate u).

The underlying reactions for the two-conformations (opening) model are given by (see Fig. S6b for a graphical representation)

$$
\begin{array}{rcl rcl rcl}
\varnothing & \xrightleftharpoons[d_T]{g_T} & T & \varnothing & \xrightleftharpoons[d_M]{g_M} & M_u & M_cP_i & \xrightarrow{e} & EM_cP_i \\
M_u & \xrightleftharpoons[]{m} & M_c & M_c & \xrightleftharpoons[c]{q\cdot o_e} & M_o & M_oP_i & \xrightarrow{e} & EM_oP_i \\
M_o, M_c & \xrightarrow{d_M} & \varnothing & T + M_c & \xrightleftharpoons[u_T]{b_T} & TM_c & EM_cP_i & \xrightleftharpoons[c]{o_i} & EM_oP_i \\
T + M_o & \xrightleftharpoons[u_T]{b_T} & TM_o & TM_c & \xrightleftharpoons[c_T]{o_T} & TM_o & EM_oP_i & \xrightarrow{u} & EM_o \\
M_o & \xrightleftharpoons[u]{b\cdot P_i} & M_oP_i & TM_o + P_i & \xrightleftharpoons[u]{b\cdot P_i} & TM_oP_i & EM_o & \xrightarrow{d_{EM}} & \varnothing \\
M_oP_i & \xrightleftharpoons[o_i]{c} & M_cP_i & TM_oP_i & \xrightleftharpoons[q\cdot o_i]{c_T} & TM_cP_i & & & \\
T + M_oP_i & \xrightleftharpoons[v_T]{a_T} & TM_oP_i & T + M_cP_i & \xrightleftharpoons[v_T]{a_T} & TM_cP_i & & &
\end{array}
$$

Using exactly the same procedure as above, we obtain the one-conformation model with

$$b \to \frac{o_e}{c + o_e} \cdot b = \hat{b} \tag{10a}$$

$$a \to \frac{q \cdot o_e}{c_T + q \cdot o_e} \cdot b = \hat{a} \tag{10b}$$

$$u_i \to \frac{o_i}{c + o_i} \cdot u \tag{10c}$$

$$q \cdot u_i \to \frac{q \cdot o_i}{c_T + q \cdot o_i} \cdot u \tag{10d}$$

Therefore, the tapasin enhancement of the peptide on-rate remains the same as for the two-conformations (unbinding) model as

$$a_r = \frac{\hat{a}}{\hat{b}} = \frac{c + o_e}{c_T + q \cdot o_e}$$

but the tapasin enhancement of the peptide off-rate becomes peptide-specific as

$$q_i = \frac{c + o_i}{\frac{c_T}{q} + o_i} = \frac{c + o_i}{x_2 + o_i}$$

We now derive an expression equivalent to (6) and (9) but cast in terms of the peptide-specific *opening* rate o_i to quantify the *filtering* achieved by this mechanism. We obtain

$$
\begin{aligned}
[EMP_i]^* \quad &= \quad \frac{o_i + c}{u \cdot o_i} \cdot \frac{e}{\frac{u \cdot o_i}{o_i + c} + e} \cdot \left(\frac{o_e}{c + o_e} \cdot b \cdot [M]^* + \frac{q \cdot o_e}{c_T + q \cdot o_e} \cdot b \cdot \frac{v_T}{v_T + \frac{q \cdot o_i}{c_T + q \cdot o_i} \cdot u} [TM]^* \right) P_i \\
&\propto \quad \frac{o_i + c}{o_i} \cdot \frac{x_1 \, (o_i + c)}{o_i + c \cdot x_1} \left([M]^* + a_r \cdot \frac{\rho \cdot o_i + \rho \cdot x_2}{o_i + \rho \cdot x_2} \cdot [TM]^* \right) \cdot P_i
\end{aligned} \tag{11}
$$

where $x_1 = \frac{e}{u+e}$, $\rho = \frac{v_T}{v_T + u}$ (< 1) and $x_2 = \frac{c_T}{q}$.

Using the relationships between the unbinding, closing and opening rates derived in this document, we can plot representative curves that compare the effective dissociation in each model (Fig. S6C). The two-conformations (unbinding) model exhibits the behaviour assumed in the one-conformation model, with tapasin increasing peptide unbinding by a constant factor. A more complex relationship was observed with the two-conformations (opening) model, with tapasin having no effect for higher values of o_i (and therefore less stable peptides). Given this fundamental difference between the two variants of the two-conformations model, it would not be unexpected that different dynamical behaviours could be observed. Therefore, we reasoned that the models would differ in their ability to reproduce experimental data when attempting to fit the underlying model parameters.

S2.2 Simulation of thermostability and endoglycosidase H resistance

All simulations were performed as done previously (*19*). The one-conformation and two-conformations (unbinding) models used representative high, medium and low affinity peptides, instantiated as three different peptide off-rates u_i. For the two-conformations (opening) model, representative peptides were instantiated with peptides for which peptide-MHC I opening o_i differed. To relate the simulations to the experimental measurements, 50°C data were compared with the high affinity peptide alone, 37°C data were compared with the sum of the high and medium affinity peptide-MHC I complexes, while 4°C data were compared with the total quantity of MHC I molecules in the system.

For endoH resistance, we considered the total quantity of egressed MHC I molecules to be *resistant*, and the intra-ER MHC I molecules to be *susceptible*. Therefore, to calculate % endoH resistance, we used the formula

$$\text{endoH} = 100 \cdot \frac{resistant}{susceptible + resistant}$$

S2.3 Bayesian parameter inference for the kinetic models

To assess the plausibility of a model of a specific circuit, we first determine optimal parameter values using probabilistic inference techniques, similar to the technique used previously (*19*). In particular, we seek to approximate the likelihood of parameters taking on specific values, given a model hypothesis and some observation data. i.e. we attempt to approximate the posterior density $Pr(\theta|H,D)$, where θ is the vector of parameters to be inferred, H is the model hypothesis, and D is the set of experimental data used for inference. The posterior distribution is related to an evidence or likelihood function $Pr(D|\theta,H)$ according to Bayes' rule:

$$Pr(\theta|H,D) = \frac{Pr(D|\theta,H)Pr(\theta|H)}{Pr(D)}$$

We obtained approximations of the posterior distributions using the Filzbach software, available from the author's website (http://research.microsoft.com/science/tools), which uses a Metropolis-Hastings (MH) Markov Chain Monte Carlo (MCMC) sampling routine. MCMC is a stochastic search strategy that forms a Markov chain of proposal parameter vectors, moving to new proposal vectors based on the ratio of the likelihoods of the proposal and previous points. By biasing the stochastic search in parameter regions of high probability mass, we converge on the true joint posterior distribution of the parameters more efficiently than would be possible with a purely random search.

To define the likelihood of a parameter vector, we assumed that the experimental measurements were noisy samples drawn independently from Gaussian distributions centered on the model predictions of the fluorescence. Therefore, in the optimal case that the model precisely describes the underlying biological behaviour, deviations of the measurements result purely from experimental error. Consequently, a data point y_i is distributed as $y_k \sim N(x_k, \sigma^2)$, where x_k is the model prediction at $t = t_k$ and σ^2 is the variance of experimental error. We assume that the variance of experimental error is proportional to the measured fluorescence signal, as quantified in Figs. 2B, 5B (i.e. $\sigma = \alpha\sqrt{y_k}$ for some α). Therefore, the likelihood function is formed as the product of the probabilities of each data-point

Table S2: The maximum likelihood parameter set for the two-conformations (opening) model with allele parameter c.

Process	Parameter	Value
MHC I supply	g_M	100.0 molecules s^{-1}
MHC I degradation in the ER	d_M	1.0534×10^{-7} s^{-1}
MHC I degradation at the cell surface	d_{EM}	5.8424×10^{-4} s^{-1}
MHC I maturation	m	$0.0021 s^{-1}$
Peptide binding	b	1.4864×10^{-6} molecules^{-1} s^{-1}
Peptide unbinding	u	1.1586 s^{-1}
Peptide availability (low)	P_1	2.26×10^5 molecules s^{-1}
Peptide availability (medium)	P_2	4.2473×10^5 molecules s^{-1}
Peptide availability (high)	P_3	1.7191×10^5 molecules s^{-1}
MHC I closing (HLA-B*44:02)	c_{02}	1.3853×10^{-6} s^{-1}
MHC I closing (HLA-B*44:05)	c_{05}	0.009 s^{-1}
MHC I closing (HLA-B*44:05^{W147A})	$c_{05.W147A}$	1.4905×10^{-4} s^{-1}
MHC I opening (peptide-free)	o_e	0.3615 s^{-1}
MHC I opening (low affinity peptide)	o_1	1.5392×10^{-4} s^{-1}
MHC I opening (medium affinity peptide)	o_2	1.4248×10^{-4} s^{-1}
MHC I opening (high affinity peptide)	o_3	1.9211×10^{-5} s^{-1}
Egression	e	3.4407×10^{-4} s^{-1}
Tapasin supply	g_T	100.0 molecules s^{-1}
Tapasin degradation	d_T	1.096×10^{-4} s^{-1}
Tapasin enhancement of opening	q	6.3616×10^3
Tapasin binding to peptide-free MHC I	b_T	4.9425×10^{-5} molecules^{-1} s^{-1}
Tapasin unbinding from peptide-free MHC I	u_T	0.0061 s^{-1}
Tapasin binding to peptide-bound MHC I	a_T	1.0164×10^{-8} molecules^{-1} s^{-1}
Tapasin unbinding from peptide-bound MHC I	v_T	0.6079 s^{-1}
MHC I closing (tapasin-bound)	c_T	0.8895 s^{-1}
Noise parameter for thermostability data	α_1	0.1046
Noise parameter for endoH data	α_2	17.3255

$$L(\theta) = Pr(D|\theta) = \prod_{k=1}^{N} \frac{1}{\alpha\sqrt{2\pi y_k}} \cdot e^{-\frac{(y_k - x_k)^2}{\alpha^2 y_k}}$$

with α left as a parameter to be inferred.

As the model simulates the concentration of peptide-MHC I complexes and not fluorescence directly, we made the simplifying assumption that there is a linear relationship between the two. Therefore, we compared the simulated output to the data with a scale factor, which was calculated using linear regression.

We determined the extent to which each model hypothesis (one-conformation, two-conformations (unbinding) and two-conformations (opening)) could reproduce experiments measuring time-dependent optimisation via thermostability (Fig. 6A) and cell surface transit via endoglycosidase H resistance (Fig. 6B). As before (*19*), we examined a variety of hypotheses for which parameters could be nominated as allele-specific, then used our parameter inference procedure to find optimal values. We then calculated the Bayesian Information Criterion (BIC), which attempts to find a compromise between having as few variable parameters as possible while minimising the deviation between model simulation and experimental observation. The BIC is defined as

$$\text{BIC} := -2 \log L(\theta^*) + k \ln(n)$$

where θ^* is the maximum likelihood estimate of the parameters (i.e the vector that maximises $L(\theta)$), k is the number of variable parameters and n is the number of experimental observations.

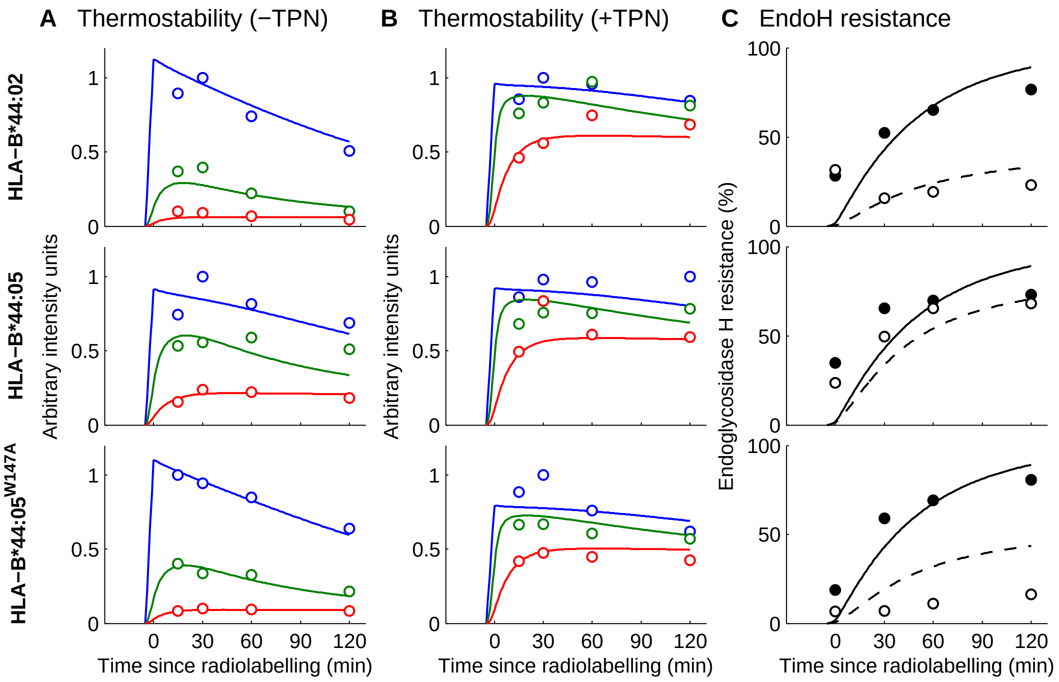

Figure S7: Comparison of best one-conformation model with experimental data. The one-conformation model with allele-specific *b* showed the best performance of all one-conformation models. The simulations and measurements are equivalent to Fig. 7B–D.

References

1. Eswar, N. *et al.* Comparative protein structure modeling using modeller. *Curr Protoc Protein Sci* **Chapter 2**, Unit 2 9 (2007).

2. Hess, B. Gromacs 4: Algorithms for highly efficient, load-balanced, and scalable molecular simulation. *Abstracts of Papers of the American Chemical Society* **237** (2009).

3. Van Der Spoel, D. *et al.* Gromacs: Fast, flexible, and free. *Journal of Computational Chemistry* **26**, 1701–1718 (2005).

4. Lindorff-Larsen, K. *et al.* Improved side-chain torsion potentials for the amber ff99sb protein force field. *Proteins* **78**, 1950–8 (2010).

5. Jorgensen, W. L., Chandrasekhar, J., Madura, J. D., Impey, R. W. & Klein, M. L. Comparison of simple potential functions for simulating liquid water. *Journal of Chemical Physics* **79**, 926–935 (1983).

6. Berendsen, H. J. C., Postma, J. P. M., Van Gunsteren, W. F. & Hermans, J. *Interaction models for water in relation to protein hydration*, vol. 331 (Reidel, 1981).

7. Hess, B., Kutzner, C., van der Spoel, D. & Lindahl, E. Gromacs 4: Algorithms for highly efficient, load-balanced, and scalable molecular simulation. *Journal of Chemical Theory and Computation* **4**, 435–447 (2008).

8. Miyamoto, S. & Kollman, P. A. Settle: An analytical version of the shake and rattle algorithm for rigid water models. *Journal of Computational Chemistry* **13**, 952–962 (1992).

9. Nose, S. A molecular dynamics method for simulations in the canonical ensemble. *Molecular Physics* **52**, 255–268 (1984).

10. Hoover, W. G. Canonical dynamics: Equilibrium phase-space distributions. *Phys Rev A* **31**, 1695–1697 (1985).

11. Parrinello, M. & Rahman, A. Polymorphic transitions in single crystals: A new molecular dynamics method. *Journal of Applied Physics* **52**, 7182–7190 (1981).

12. Nose, S. & Klein, M. L. Constant pressure molecular dynamics for molecular systems. *Molecular Physics* **50**, 1055–1076 (1983).

13. Essmann, U. *et al.* A smooth particle mesh ewald method. *Journal of Chemical Physics* **103**, 8577–8593 (1995).

14. Humphrey, W., Dalke, A. & Schulten, K. Vmd: Visual molecular dynamics. *Journal of Molecular Graphics* **14**, 33–& (1996).

15. Pettersen, E. F. *et al.* Ucsf chimera - a visualization system for exploratory research and analysis. *Journal of Computational Chemistry* **25**, 1605–1612 (2004).

16. Young, M. A., Gonfloni, S., Superti-Furga, G., Roux, B. & Kuriyan, J. Dynamic coupling between the sh2 and sh3 domains of c-src and hck underlies their inactivation by c-terminal tyrosine phosphorylation. *Cell* **105**, 115–26 (2001).

17. Amadei, A., Linssen, A. B. & Berendsen, H. J. Essential dynamics of proteins. *Proteins* **17**, 412–25 (1993).

18. Hub, J. S. & de Groot, B. L. Detection of functional modes in protein dynamics. *Plos Computational Biology* **5** (2009).

19. Dalchau, N. *et al.* A peptide filtering relation quantifies MHC class i peptide optimization. *PLoS Comput Biol* **7**, e1002144 (2011).
